# Magnetic manipulation of the reactivity of singlet oxygen: from test tubes to living cells

**DOI:** 10.1093/nsr/nwae069

**Published:** 2024-02-27

**Authors:** Zi-Shu Yang, Song Gao, Jun-Long Zhang

**Affiliations:** Institute of Inorganic Chemistry, College of Chemistry and Molecular Engineering, Peking University, Beijing 100871, China; Institute of Inorganic Chemistry, College of Chemistry and Molecular Engineering, Peking University, Beijing 100871, China; Spin-X Institute and Guangdong-Hong Kong-Macao Joint Laboratory of Optoelectronic and Magnetic Functional Materials, South China University of Technology, Guangzhou 510641, China; Institute of Inorganic and Material Chemistry, School of Chemistry and Chemical Engineering, Sun Yat-sen University, Guangzhou 510275, China; Chemistry and Chemical Engineering Guangdong Laboratory, Shantou 515031, China; Institute of Inorganic Chemistry, College of Chemistry and Molecular Engineering, Peking University, Beijing 100871, China; Chemistry and Chemical Engineering Guangdong Laboratory, Shantou 515031, China

**Keywords:** oxygen metabolism, spin regulation, magnetic field effect, radical pair mechanism

## Abstract

Although magnetism undoubtedly influences life on Earth, the science behind biological magnetic sensing is largely a mystery, and it has proved challenging, especially in the life sciences, to harness the interactions of magnetic fields (MFs) with matter to achieve specific ends. Using the well-established radical pair (RP) mechanism, we here demonstrate a bottom-up strategy for the exploitation of MF effects in living cells by translating knowledge from studies of RP reactions performed *in vitro*. We found an unprecedented MF dependence of the reactivity of singlet oxygen (^1^O_2_) towards electron-rich substrates (**S**) such as anthracene, lipids and iodide, in which [**S**^˙+^ O_2_^˙−^] RPs are formed as a basis for MFs influencing molecular redox events in biological systems. The close similarity of the observed MF effects on the biologically relevant process of lipid peroxidation in solution, in membrane mimics and in living cells, shows that MFs can reliably be used to manipulate ^1^O_2_-induced cytotoxicity and cell-apoptosis-related protein expression. These findings led to a ‘proof-of-concept’ study on MF-assisted photodynamic therapy *in vivo*, highlighting the potential of MFs as a non-invasive tool for controlling cellular events.

## Teaser

A bottom-up approach was developed for magnetic regulation of oxygen metabolism related cellular activities via magnetic-field-dependent ^1^O_2_ reactivity.

## INTRODUCTION

In parallel with advances in research on animal magnetoreception over the last few decades [1–6], molecular mechanisms have emerged for magnetic field (MF) effects (MFEs) in chemistry. In the 1970s, the radical pair (RP) mechanism was proposed as a route by which applied MFs could alter the yields of free radical reactions, enabling scientists to use MFEs to interrogate chemical systems [5,7]. However, its translation to biological systems has proved challenging; in particular, it has yet to be established how MFs can be used to manipulate cellular activity [8,9]. One of the major obstacles has been the difficulty in getting reproducible experimental data from complex cellular systems that naturally comprise paramagnetic metal ions and a variety of free radical species [10,11]. Here, we aim to establish an interdisciplinary, bottom-up approach to guide MF studies of living cells by the introduction of RP reactions and by translating insights obtained *in vitro* in order to avoid endogenous interference.

**Figure 1.**
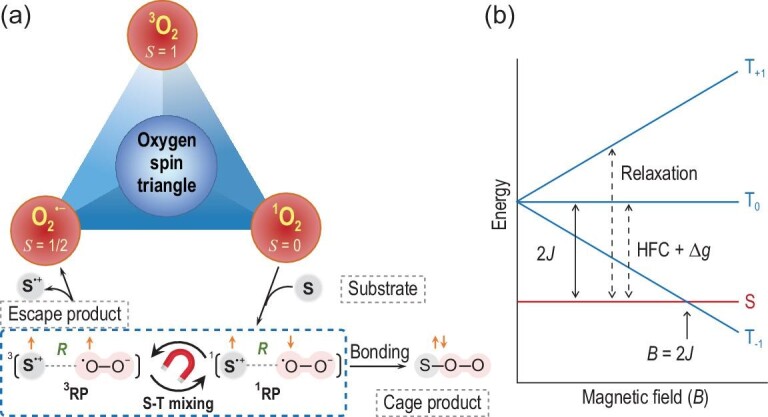
Illustration of the foundations for MFE. (a) The spin representation and the interconversion of oxygen and primary ROS (^1^O_2_ and O_2_^˙−^) in an ‘oxygen spin triangle’, and an illustration of two fundamental spin selective reaction mechanisms of ^1^O_2_, which could be magnetic sensitive: generation of a radical pair (RP) or biradical (BR) by electron transfer from a substrate **S**. *R* represents the distance between the radical centers. (b) Zeeman splitting of the spin energy levels of an RP or a BR. 2*J* is the exchange interaction of two electrons (*J* can be negative or positive; *J* < 0 in the figure). *a*_eff_ is the effective hyperfine interaction of the electrons with nuclear spins. Singlet-triplet (S-T) mixing can be induced by spin relaxation, hyperfine coupling, the ‘2*J*-resonance’ at an applied magnetic field *B* = 2*J*, and the difference in the *g*-factors (Δ*g*) of the two radicals. The energy scale is arbitrary.

Reactive oxygen species (ROS) in oxygen metabolism are primarily a group of molecules derived from molecular oxygen. Control of three-spin states of ROS represented in an ‘oxygen spin triangle’, as shown in Fig. 1a, allows for manipulating ROS reactivities from the perspective of spin angular momentum in principles such as electron spin-flip by light irradiation (photochemistry) or spin-state switching with redox active metal/organic interfaces (electrochemistry), rather than being solely governed by reaction thermodynamics. Based on the quantum nature of RP, herein we introduce a tunable modulation of ^1^O_2_ reactivity by MFs (magnetochemistry) and hence the yields of singlet (cage) and triplet (escape) reaction products. Singlet oxygen, ^1^O_2_ (^1^Δ_g_), is an excited state of O_2_ in which the highest occupied molecular orbitals (of π_g_ symmetry) contain two electrons with antiparallel spins. ^1^O_2_ readily oxidizes cellular components (substrates, **S**) such as lipids, proteins and nucleic acids [12,13]. Such oxidations render the formation of a weakly coupled RP ([**S**^˙+^ O_2_^˙−^]) or a biradical (BR, ^˙^**S**–O–O^˙^) via one-electron transfer (Fig. 1a) [14]. Within the framework of the RP mechanism, the application of an MF produces a Zeeman splitting of the triplet sublevels (T_0_, T_±1_) of both [**S**^˙+^ O_2_^˙−^] and ^˙^**S**–O–O^˙^ (Fig. 1b), leading to field-dependent interconversion of the singlet and triplet electron-spin states. MFEs arise from different mechanisms that depend on the electron exchange interaction (2*J*), the effective electron-nuclear hyperfine coupling (HFC) of the two radicals (*a*_eff_), the difference in their *g*-factors (Δ*g*), and spin relaxation [15], allowing scope for modulation of ^1^O_2_ reactivity.

Supposing that a living cell can be viewed as a reaction vessel containing a set of substrates in the viscous cytosol, one could imagine introducing ^1^O_2_ so as to permit magnetic manipulation of oxidative stress, especially under photo-irradiation conditions where the action of ^1^O_2_ would overwhelm interference arising from endogenous ROS fluctuations. This would open the possibility of bottom-up magnetic regulation of cellular activity via MF-dependent redox homeostasis driven by molecular events.

Here, we seek to implement the RP mechanism, as described above, beginning with the reactions of ^1^O_2_ with various substrates, including iodide anion, anthracenes and lipids using MFs in the range 0–800 mT. Having established MFs as a reliable tool for modulating ^1^O_2_ reactivity, we focus on biologically relevant lipid peroxidation and then correlate the MFEs occurring in solution with those in living cells. This approach extends our understanding of MFEs on redox homeostasis and informs the relationship between ^1^O_2_-induced cell photocytotoxicity, cell apoptosis and apoptosis-induced biomarker proteins. Finally, we present an *in vivo* ‘proof-of-concept’ demonstration that external MFs synergistically facilitate photodynamic therapy (PDT) which uses ^1^O_2_ to kill cancer cells, highlighting the potential of the RP mechanism for magneto-medical purposes.

## RESULTS AND DISCUSSION

### MFs modulate ^1^O_2_ reactivity

Previously, Boxer [16], Mathis [17] and Hore [18] have reported MFEs on ^1^O_2_ generation using photosensitizers that have excited states with significant charge-transfer characteristics. To minimize MF-dependent formation of ^1^O_2_, we chose Rose Bengal (RB) and chlorin e6 (Ce6) as photosensitizers (PSs) (Fig. S1a), whose photophysical properties, including ^1^O_2_ photosensitization, showed less than ±10% variation in the MF range of 0–1000 mT (Fig. S1b–d).

**Figure 2.**
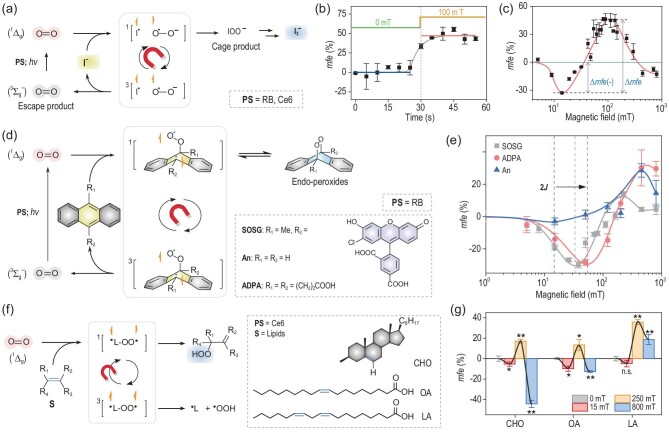
MFE on the reactions of ^1^O_2_. (a) Mechanism of the reaction of ^1^O_2_ with I^−^, which generates the RP intermediate [I^˙^ O_2_^˙−^] and gives rise to an MFE on the reaction rate. (b) Average *mfe* on the generation rate of I_3_^−^ (*mfe* = (*r*_B_ − *r*_0_)/*r*_0_ × 100%) for four on-off steps calculated from the differential of the data in Fig. S3. The red and blue lines give the mean *mfe* values in the field-on and field-off periods. (c) Dependence of the *mfe* values on the MF strength for the reaction with I^−^. (d) The BR pathway of cycloaddition and the thermolysis of the corresponding endoperoxide as the reverse reaction. Box: structures of the anthracenes. (e) MFE on the reaction rate for the oxidation of SOSG (square), ADPA (circle) and An (triangle) as a function of applied MF. RB: 10 μM; KI: 10 mM, anthracene derivatives: 10 μM; irradiated at 561 nm, 5 mW cm^−2^. (f) The BR pathway of C=C lipid (**L**) peroxidation by ^1^O_2_. Box: structures of lipids used in this work. (g) MFE on the conversion (*Conv, mfe* = (*Conv*_B_ − *Conv*_0_)/*Conv*_0_ × 100%) for the reaction with lipids in CHCl_3_-*d*. Lipids: 0.05 mmol, photosensitizer = Ce6: 0.5 mol%; irradiation condition: 635 nm, 20 mW cm^−2^, 1 h, air atmosphere. Conversions were determined by ^1^H NMR analysis using 1,1,2,2-tetrabromoethane as an internal standard. Data presented as mean ± SD (*n* = 3). *P < 0.05, **P < 0.01.

We began with oxidation of an iodide anion (I^−^) by ^1^O_2_, in which we propose that a [I^˙^ O_2_^˙−^] RP is formed by one-electron transfer (Fig. 2a) [19]. The reaction rate (*r*) was estimated based on the changes in I_3_^−^ absorption at 350 nm (Fig. S2a). The fractional MFE is defined as *mfe* = [*r*_B_ − *r*_0_)]/*r*_0_ × 100%, where *r*_B_ and *r*_0_ are the rates determined in the presence and absence of an MF (strength *B*; see Methods for details), respectively. Prior to the MFE study, we performed control experiments to determine whether I_3_^−^ is generated in the absence or presence of O_2_ under irradiation, and found that no reaction occurred (Fig. S2b). To test for MFEs, we examined the reaction rate for periodically switched MFs (100 mT). As shown in the differential absorbance-time plot (Fig. S3), an apparent abrupt *r* enhancement with an average *mfe* of ca. 47% in a 100 mT field was observed (Fig. 2b), verifying the MF-dependence of I^−^ oxidation by ^1^O_2_.

Then, we measured the *mfe*s by varying the applied MF in the range of 0–800 mT and plotted *mfe* (%) vs. field strength as shown in Fig. 2c and Fig. S4. When the MF was increased from 0 to 14 mT, a negative effect, Δ*mfe*(−), was observed with a maximum amplitude of −33% at 14 mT. We ascribe this negative MFE to the low field effect (LFE), i.e. the lifting of energy-level degeneracies by the Zeeman interaction, which promotes singlet-triplet (S-T) mixing [20]. Above 14 mT, the *mfe* values increased to a positive maximum of 46% at 130 mT, a change we attribute to the energetic isolation of the T_±1_ sublevels by the Zeeman interaction, which suppresses the ${\mathrm{S}} \to {{{\mathrm{T}}}_{ \pm 1}}$ transitions induced by the HFCs. Thus, HFC-driven S-T_0_ interconversion comes to dominate as the field is increased, accounting for the positive MFE. On increasing the MF to 800 mT, the measured *mfe* fell back to −13%, an effect we interpret as due to the increased S-T_0_ interconversion arising from the difference in the Zeeman interactions of I^˙^ and O_2_^˙−^ arising from Δ*g*. To summarize, across the range of MFs from 0 to 800 mT, we observed a ‘down-up-down’ trend in the MFE reflecting the extent of S-T mixing arising from the LFE, HFC and Δ*g* mechanisms. The MFEs on this reaction are large, with a total Δ*mfe* (defined in Fig. 2c) of 79%. When using Ce6 as PS, similar down-up-down MF dependence was observed with negative/positive maximum MFE of −20% (15 mT) and 27% (90 mT), respectively (Fig. S5). The result supports our hypothesis that the magnetic sensitivity stems from the reactivity of ^1^O_2_ rather than the photosensitization process. This behavior can be simulated using the spin-dynamics methods described in ref. [21] (Supplementary Data). These relatively simple calculations, based on the LFE-HFC-Δ*g* mechanisms, reproduce the shape of the experimental field-dependence although they have trouble predicting a Δ*mfe* as large as 79% (Fig. S6).

Having demonstrated that MFs can influence the recombination rate of [I^˙^ O_2_^˙−^], we next targeted a BR where the two unpaired electrons are in the same molecule. To this end, we identified anthracene peroxidation as a model reaction involving a [^˙^R–OO^˙^] BR (Fig. 2d); this is the reverse reaction of the magnetically sensitive thermolysis of endoperoxides reported by Turro *et al.* [22]. We began our endeavor by investigating the oxidation of Singlet Oxygen Sensor Green^®^ (SOSG), a commercially available ^1^O_2_ probe, using an emission intensity of 525 nm (Fig. S7). As shown in Fig. 2e, from 0 to 30 mT negative *mfe*s were found with a maximum effect of −30% at 34 mT, followed by an increase between 34 and 250 mT with a maximum of 14% at 250 mT. Stronger fields, up to 800 mT, reduced *mfe* to 4.5%. A total ∆*mfe* of 44% was observed. Due to the short distance and rigid link between the intramolecular radical centers, 2*J* in this case is likely to be large compared to *a*_eff_, implying that S-T_±1_ mixing increases progressively and dominates when the MF is approximately equal to 2*J*. Thus, we tentatively estimated 2*J* to be 34 mT, which is larger than typical *a*_eff_ values (1–10 mT) of organic BRs. At higher MFs, suppression of spin relaxation leads to an increase in *mfe*, followed by the decrease resulting from the Δ*g* mechanism. When anthracene (An) and anthracene-9,10-dipropionic acid (ADPA) were used as substrates, similar MF-dependence was found (Fig. 2e and Figs S7 and S8). However, the substituents significantly impact 2*J* and ∆*mfe*, probably arising from the intramolecular interactions of the two electron spins [23,24] or different reaction pathways [22]. Nevertheless, despite the different (RP or BR) intermediates, our results demonstrate that these ^1^O_2_-mediated oxidations are all MF-sensitive and exhibit the same down-up-down pattern of MF-dependence as seen for ^1^O_2_ and I^−^.

The cellular membrane, featuring lipid analogs assembled into a continuous bilayer structure, is an important organelle and barrier; lipid peroxidation by ^1^O_2_ is believed to trigger deleterious membrane damage and to initiate cell apoptosis [25]. We envisaged that lipid peroxidation forming BR-type intermediates might serve as a critical bridge to correlate the MFEs observed in test tubes and in living cells. Prior to cellular study, we chose cholesterol (CHO, Fig. 2f, and Fig. S11), an essential component of plasma membranes, as a substrate. MFEs were estimated by comparing the conversion of lipids in 15, 250 and 800 mT MFs, corresponding to the different mechanisms mentioned above. As shown in Fig. 2g, *mfe*s of −5.3%, 17% and −44% were measured at the mentioned MF strengths, respectively. Next, linear unsaturated fatty acids such as oleic acid (OA) and linoleic acid (LA) were investigated. *mfe*s of −9.8%, 13% and −13% for OA, and −5.0%, 36% and 19% for LA were obtained at 15, 250 and 800 mT, respectively (Fig. 2g, and Fig. S12). Thus ^1^O_2_-induced lipid peroxidation is MF-dependent, with the detailed effects depending on structural factors, such as the unsaturation level of the lipid, and steric effects.

### Correlating MFEs in test tubes and in living cells

**Figure 3.**
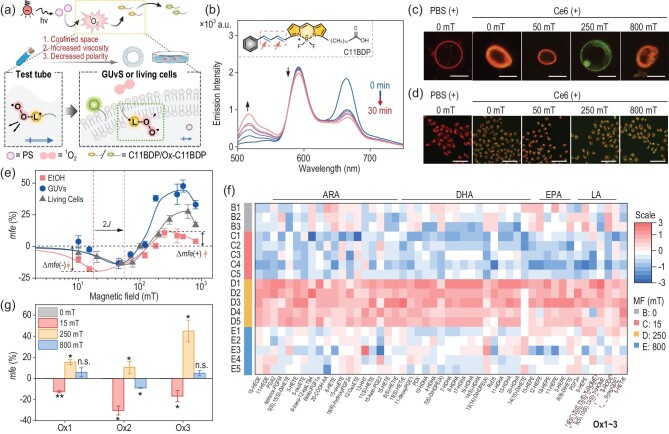
MFE on the lipid peroxidation by ^1^O_2_ in solution and living cells. (a) Illustration of the photo-oxidation of C11BDP in a test tube (EtOH solution), GUVs or living cells. (b) Emission spectra of C11BDP in the presence of Ce6 in EtOH recorded as a function of time in the absence of applied MF. Inset: structure of C11BDP. (c, d) Representative fluorescence images of GUVs (c) and HeLa cells (d) treated with phosphate-buffered saline (PBS, pH = 7.4) or Ce6 and stained with C11BDP, and irradiated (10 min) in different external MFs (0, 50, 250, and 800 mT). (e) MFE on the oxidation level of C11BDP in different conditions using the intensity ratio of oxidized-/original-C11BDP probe (*R* = *I*_Green_/*I*_Red_): *mfe* = (*R*_B_ − *R*_0_)/*R*_0_ × 100%. Ce6: 20 μM, C11BDP: 20 μM. Irradiation condition: 635 nm; for experiments in EtOH solution: 20 mW cm^−2^; in GUVs and cells: 5 mW cm^−2^, 10 min. ‘+’ indicates photo-irradiation. (f) Heat map of the content change of oxylipids identified for Groups A–E using Caki-1 cells (*n* = 3 for Group A and B, *n* = 5 for Groups C–E). ARA: arachidonic acid, DHA: docosahexaenoic acid, EPA: eicosapentaenoic acid. (g) MFE on the level of representative oxylipids as LA oxidative metabolites. Cells in Groups A–E were treated with PBS (A) or RB (B–E, 20 μM, 12 h) and received irradiation in 0 (A and B), 15 (C), 250 (D) and 800 mT (E) MFs. Ox1–3 are shown in Fig. 3f. Irradiation conditions for oxidative lipidomics: 400–700 nm white light, 5 mW cm^−2^, 10 min. Data are presented as mean ± SD. *P < 0.05, **P < 0.01, n.s.: no significance.

To compare MFEs on lipid oxidation in different environments, such as ethanol (EtOH) solution, giant unilamellar vesicles (GUVs) as mimics of cell membranes [26], and living cells (Fig. 3a), we used the fluorescent probe C11-BODIPY^581/591^ as a lipid surrogate (C11BDP, Fig. 3b and Fig. S13a) [27], allowing real-time monitoring of lipid peroxidation via its ratiometric fluorescence change (Fig. S13b). To avoid interference from the absorption and emission of PS, Ce6 were used instead of RB. In GUVs and cells, lipid oxidation was investigated by confocal fluorescence microscopy, displaying the MF-regulated oxidation level through changes in green and red fluorescence intensities in GUVs (Fig. 3c and Fig. S14) and living cells in different MFs (Fig. 3d and Fig. S15). As shown in Fig. 3e, a similar down-up-down trend of MF dependence, based on the combination of 2*J*-resonance/spin relaxation/Δ*g* mechanisms, was obtained from the ratio of green/red fluorescence intensities in the three experimental conditions. In EtOH, the *mfe* fell to −21% at 22 mT followed by a plateau at 250 mT with a maximum *mfe* of 11%. Above 250 mT, the *mfe* decreased. Comparison of MFEs measured in EtOH, GUVs and living cells manifested effects of the environment on the spin-state mixing of the BR. For example, at low MFs, the field strength corresponding to the maximum negative *mfe* (tentatively assigned to 2*J*) gradually shifted to higher fields (from 22 mT to 50 mT) in the order EtOH < GUVs ≈ living cells, accompanied by a decrease in ∆*mfe*(−) (from ca. 21% to 14%) and an increase in ∆*mfe*(+) (from ca. 11% to 28% and 44%). Considering that 2*J* strongly depends on the energy of the BR [28], we suppose that compared with the EtOH solution, both GUVs and living cell membranes provide a less polar environment, which results in a larger 2*J* (Fig. 3a). In addition, the constrained space may enhance spin relaxation and shorten RP lifetimes [29], resulting in a larger ∆*mfe*(+) but smaller ∆*mfe*(−) in GUVs and cells. The close correlation of MFEs in different media is critical for translating insights obtained from test-tube experiments to establish a guide for harnessing cellular activity using applied MFs.

To further visualize the cellular reactivity of ^1^O_2_ on lipids, we performed targeted oxidative lipidomics analysis. Caki-1 cells were used due to their high cytosolic lipid content. A heat map was computed using control groups (Group A) as reference and including all 94 detected oxidative lipid metabolites (Fig. S16). To better address the MFE, the groups with no applied MF as reference were used, and restricted to oxylipids whose concentration changed statistically significantly between 0 (Group B) and 250 mT (Group D, *P* < 0.05) (Fig. 3f). Generally, the content of oxylipids increased in 250 mT, while it decreased among those exposed to either 15 (Group C) or 800 mT (Group E). Specifically, we examined the level of four main differential metabolites in LA oxidation metabolism, and found an approximately down-up-down regulation for low-moderate-high MF, with Δ*mfe* up to 62% (Fig. 3g). The MF dependence is similar to the reactivity in solution, confirming that MFs influence cellular events via ^1^O_2_.

### MFs rationally regulate ^1^O_2_-induced cytotoxicity

**Figure 4.**
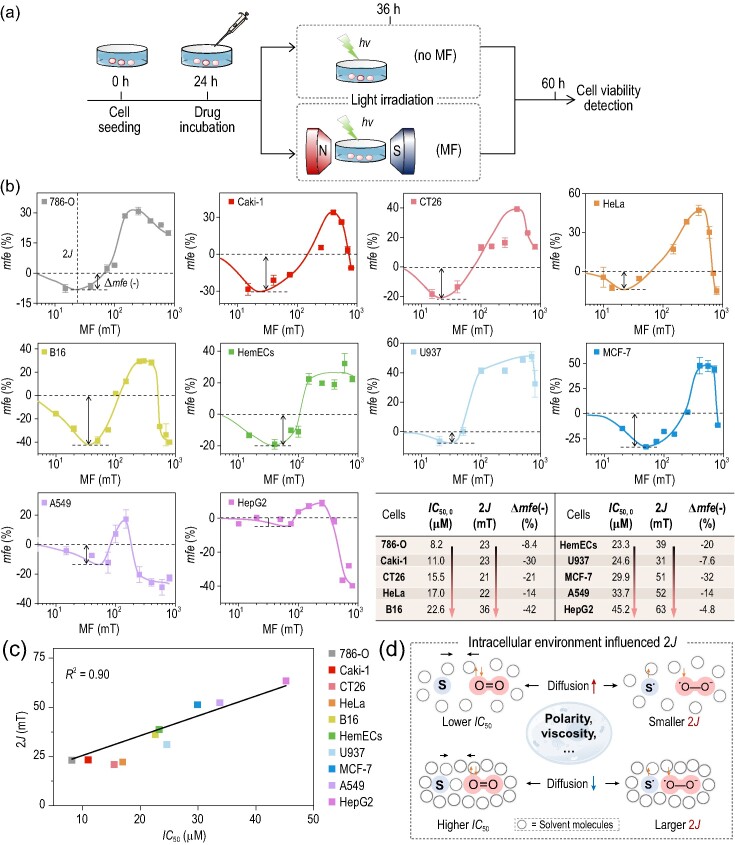
MFE on *in vitro* cytotoxicity in living cells. (a) Illustration of the procedure for *in vitro* photocytotoxicity against cancer cells. (b) MF-dependence of photocytotoxicity for different cell lines (786-O, Caki-1, CT26, HeLa, B16, HemECs, U937, MCF-7, A549 and HepG2) and a summary of the *IC*_50, 0_, 2*J* and Δ*mfe*(−) values. *mfe* = –(*IC*_50, B_ − *IC*_50, 0_)/*IC*_50, 0_ × 100%. Data are presented as mean ± SD (*n* = 3). A positive *mfe* indicates enhanced inhibition of cell proliferation, whereas a negative value means reduced photocytotoxicity. (c) Dependence of 2*J* values on *IC*_50,0_. (d) Illustration of the influence of the intracellular environment on MFEs. PS = RB: 20 μM. Irradiation condition: 400–700 nm white light, 5 mW cm^−2^, 10 min.

That ^1^O_2_ is one of the critical ROS responsible for initiating cell cytotoxicity in PDT [30], and provides an opportunity to investigate MFEs on cellular activity. ^1^O_2_-induced cytotoxicity arising from irradiation of RB *in situ* after incubation in cells was examined in the presence of 0–800 mT MFs (Fig. 4a). The *mfe* on cytotoxicity is defined as the reduction in the half-inhibitory concentration (*IC*_50_) in an MF of strength *B* compared to that obtained with no applied MF. As shown in Fig. S17 and Fig. 4b, increasing the MF from zero to 14 mT led to a decrease in cytotoxicity with an *mfe* of −14%. From 14 to 400 mT, enhanced cytotoxicity was observed with a maximum positive *mfe* of 48%. Increasing the MF from 400 to 800 mT produced a decrease in *mfe*, to −15% at 800 mT. Δ*mfe* in the experimental MF range was ca. 48%. The control groups in the dark showed minor cytotoxicity toward HeLa cells, whether in the absence or presence of MFs (Fig. S18). The experiments were also carried out using Ce6 as PS. MFE on *IC*_50_ values are found to be −35%, 4.8% and −28% for 15, 250 and 800 mT MF, respectively (Fig. S19), extending the scope of PSs. To examine the generality of this observation, we tested nine other cancer cell lines from different body locations, including 786-O, Caki-1, CT26, B16, HemECs, U937, MCF-7, A549 and HepG2. All of these cell lines displayed a similar down-up-down trend of MF-dependence of cytotoxicity, featuring the MFE based on LFE/2*J*-resonance-HFC/spin relaxation-Δ*g* mechanisms, clearly showing the feasibility of using MFs to modulate ^1^O_2_-mediated cell viability (Fig. 4b).

The observed MFEs varied from cell to cell, for example in the low field region (0–100 mT), the values of Δ*mfe*(−) were −8.2%, −30%, −21%, −14%, −43%, −20%, −7.6%, −31%, −14% and −4.8% for the mentioned cell lines, respectively, independent of the *IC*_50_ values (Fig. S20). However, the 2*J* parameter was found to be negatively related to cytotoxicity as revealed by plotting 2*J* against *IC*_50_ (Fig. 4c), showing MF-sensitivity at the cellular level. Concerning the magnetic control of the oxidation of C11BDP discussed above (Fig. 3a), 2*J* is the magnitude of the exchange interaction between the electron spins of RPs in cellular cytosols characterized by high viscosity, polarity or confined spaces, which exert a profound influence on the chemical kinetics (Fig. 4d) [31]. Meanwhile, slower diffusion might reduce the encounter possibility between ^1^O_2_ and biomolecules, which could contribute to the reduced cytotoxicity [32]. It is difficult to rationalize the exact reasons for the changes in 2*J* and Δ*mfe* in different cell lines due to the complexity of these living systems; however, our results suggest that MFs exert a profound influence at cellular levels.

**Figure 5.**
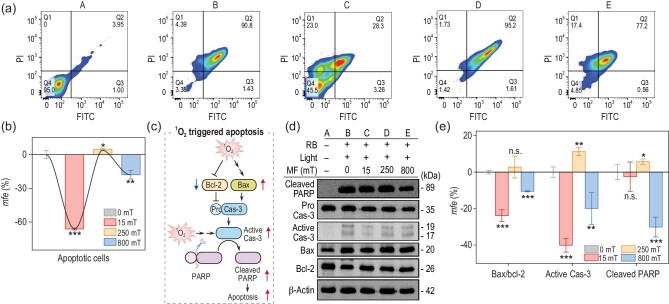
MFE on the ^1^O_2_-induced apoptosis against HeLa cells. (a) Representative flow cytometry analysis of HeLa cells stained using the Annexin V-FITC/PI apoptosis assay. The quadrants from the lower left to the upper left (counterclockwise) represent healthy (Q4), early apoptotic (Q3), late apoptotic (Q2) and necrotic cells (Q1), respectively. (b) Quantification of the MFEs on apoptosis using the amounts (*C*) of apoptotic cells (Q2 + Q3). *mfe* = (*C*_B_−*C*_0_)/*C*_0_ × 100%. (c) Illustration of the ^1^O_2_-induced apoptosis signaling pathway mediated by representative proteins (Bcl-2, Bax, pro-/active Cas-3 and cleaved PARP). (d) Western blot analysis of apoptosis-related proteins in HeLa cells. (e) Quantification of the MFE on the levels of Bax/Bcl-2 ratio, active Cas-3 and cleaved PARP, calculated by the integrated density (*D*) in (d) using Image J. Data were normalized to β-Actin. *mfe* = (*D*_B_−*D*_0_)/*D*_0_ × 100%. Cells in Groups A–E were treated with PBS (A) or RB (B–E, 20 μM, 12 h) and received irradiation in 0 (A and B), 15 (C), 250 (D) and 800 mT (E) MFs. Cells were collected for flow cytometry or western blot analysis 24 h post-irradiation. Irradiation conditions: 400–700 nm white light, 5 mW cm^−2^, 10 min. Data in (b) and (e) are presented as mean ± SD (*n* = 3). *P < 0.05, **P < 0.01, ***P < 0.005, n.s.: no significance.

We further characterized MFEs on ^1^O_2_-induced cell death pathways using flow cytometry with the Annexin V Apoptosis Detection Kit. As shown in Fig. 5a and b, compared with the control group with no applied MF, exposure to a 250 mT MF led to an increase of 5% in apoptotic cells, in contrast to decreases of 66% and 18% at 15 and 800 mT, respectively. These results indicate that a moderate MF (250 mT) activates apoptosis, while low (15 mT) or high (800 mT) MF has the opposite effect. Next, we examined the expression of apoptosis-related proteins, such as B-cell lymphoma-2 (Bcl-2), Bcl-2-Associated X protein (Bax), Caspase-3 (Cas-3) and poly ADP-ribose polymerase (PARP) (Fig. 5c) [33]. As shown in Fig. 5d and e and Fig. S21, compared with the control group (Group B), the Bax/Bcl-2 ratio increased to 3.2% at 250 mT (Group D), but decreased to −24% (Group C) and −11% (Group E) at 15 and 800 mT, respectively, suggesting the promotion of apoptosis. Similar MF-dependent active Cas-3 and PARP levels were observed. These results suggest that the ^1^O_2_-induced apoptosis rate is also magnetically controlled.

### MFs regulate ^1^O_2_-induced *in vivo* PDT efficacy

**Figure 6.**
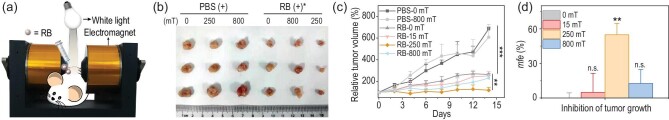
MFE on *in vivo* PDT efficacy. (a) Illustration of PDT treatment for HeLa-tumor-bearing mice in an MF. (b) Photographs of retrieved tumor tissues in different mice groups at day 14. *Tumor placed in the order of tumor size instead of the experimental MF magnitude. (c) Tumor growth profiles for different mouse groups. (d) MFE on the inhibition effect of tumor growth based on the average volume (*V*) of tumor tissues in (c) at day 14. *mfe* = –(*V*_B_−*V*_0_)/*V*_0_ × 100%. Data are presented as mean ± SD (*n* = 9). PBS or RB (1.0 mg kg^−1^) were intratumorally injected. All groups received photo-irradiation 5 min post-injection, 400−700 nm white light, 100 mW cm^−2^, 10 min. **P < 0.01, ***P < 0.005, n.s.: no significance.

To extend the insights into ^1^O_2_ reactivity obtained at the cellular level, we attempted to use MFs to manipulate the efficacy of anti-tumor PDT *in vivo* using a total of 81 HeLa tumor-bearing mice randomly divided into nine groups: (1) phosphate-buffered saline (PBS)-dark, (2) PBS (no MF), (3) PBS-15 mT, (4) PBS-250 mT, (5) PBS-800 mT, (6) RB (no MF), (7) RB-15 mT, (8) RB-250 mT and (9) RB-800 mT. After intratumoral injection of RB (1.0 mg kg^−1^) or equivoluminal PBS, all groups were photo-irradiated (400−700 nm, 100 mW cm^−2^, 10 min) 5 min post-injection, except Group (1). The tumor regions of mice in Groups (3)–(5) and (7)–(9) were additionally exposed to an MF using an electromagnet during photo-irradiation (Fig. 6a). As shown in Fig. 6b–d and Figs S22 and S23, two weeks after treatment (day 14), the PDT group (Group (6)) showed inhibition of tumor growth, which was further suppressed by 55% in a 250 mT MF (Group (8)). Both a weak field (15 mT, Group (7)) and a strong field (800 mT, Group (9)) had a small positive effect on the efficacy (5.0% and 13%, respectively) but without statistical significance according to Student's *t*-test. These results were further verified by a 71% decrease in the average weight (*ω*) of retrieved tumor tissues found for PDT at 250 mT (Group (6)) compared with that of Group (5) (0 mT, Fig. 6b). Therefore, a moderate MF around 250 mT can strongly promote *in vivo* PDT efficacy, revealing the potential of applying MFs to enhance the *in vivo* inhibition of tumor growth by combination with PDT.

## DISCUSSION

Radicals occur naturally throughout biology [34] and their chemical reactions are known to be sensitive to applied magnetic fields. It is therefore of interest to ask whether magnetic fields could be used to modulate the generation and transformation of radicals (in particular ROS radicals) *in vivo*, which is also important for understanding the chemical origin of life. In contrast to the well-established MF-modulation of charge-separation/recombination processes in quantum sensing [31], luminescence [35] and photovoltaics [36], there is no reliable guide for rational magnetic manipulation of redox homeostasis in living cells [37]. Mermut *et al.* [38] used a magneto-optical PS to manipulate ^1^O_2_ yield with MF, and observed a 50% increase in cell survival in PDT treatment. However, the effect is largely dependent on the structure and photophysical properties of PS. In this work, we introduce a bottom-up approach starting from the MF-dependent reactivity of ^1^O_2_ toward various substrates such as iodide, anthracenes and lipids. Consistent with the RP mechanism, the initial singlet RP [**S**^˙^ O_2_^˙−^] or BR (^˙^**S**–O–O^˙^) formed from ^1^O_2_ and an electron-rich substrate shows a down-up-down MF-dependence in low (0–30 mT), moderate (30–400 mT) and high (400–800 mT) MFs, respectively, corresponding to the LFE, HFC and Δ*g* mechanisms for weakly coupled RPs. In the case of more rigidly linked BR intermediates, the low field region is governed by 2*J*-resonance. Incoherent relaxation [39] and spin-orbit coupling [40] might also contribute as a quencher of MFE, resulting in a smaller magnitude of MFE and broader linewidth of magnetically affected reaction yield (MARY) curve. By correlating MFEs in EtOH solution, GUVs and living cells, it is clear that the medium parameters, such as viscosity [41] and polarity [42], have a strong impact on the magneto-sensitivity, including the response range and magnitude. This indicates that it is feasible to realize magnetic manipulation in living cells based on the MFEs observed under non-physiological conditions.

MFEs on cell photocytotoxicity, in which ^1^O_2_ was considered the active species, exhibited a similar MF dependence to those observed in solution. This encouraged us to use MFs as a general strategy to modulate cytotoxicity in 10 cell lines and expression of apoptosis-related biomarkers. We found that exposure to low and high MF strengths (e.g. 15 and 800 mT) provokes an antagonistic effect, while a synergistic enhancement was observed at moderate MF strengths (e.g. 250 mT). The consistent MF dependence in the accumulation of oxylipids analyzed by oxidative lipidomes and the oxidation reactivity of ^1^O_2_ toward lipids in solution, demonstrated that the RP mechanism provides an explicit framework for using MFs to up- or down-regulate ^1^O_2_-induced molecular events or redox homeostasis in living systems. This work provides an unprecedented bottom-up paradigm for the rational use of MFs for biomedical purposes as a non-invasive tool, by transplanting a non-native chemical reaction with significant MFE into biological processes.

## Supplementary Material

nwae069_Supplemental_File
