# Supplementary material for: Magnetic manipulation of the reactivity of singlet oxygen: from test tubes to living cells
Source: Natl Sci Rev. 2024 Feb 27;11(9):nwae069. doi: 10.1093/nsr/nwae069 (PMC11321247; doi:10.1093/nsr/nwae069)
Supplement: nwae069_Supplemental_File [file nwae069_supplemental_file.docx]

Supplementary Data for

**Magnetic Manipulation of the Reactivity of Singlet Oxygen: From Test Tubes to Living Cells**

Zi-Shu Yang^1^, Song Gao^1,2,3,4^* & Jun-Long Zhang^1,4^*

Corresponding author: [gaosong@pku.edu.cn](mailto:gaosong@pku.edu.cn) (S. G.); [zhangjunlong@pku.edu.cn](mailto:zhangjunlong@pku.edu.cn) (J.-L. Z.)

^1^College of Chemistry and Molecular Engineering, Peking University, Beijing 100871, P. R. China.

^2^Spin-X Institute and Guangdong-Hong Kong-Macao Joint Laboratory of Optoelectronic and Magnetic Functional Materials, South China University of Technology, Guangzhou 510641, China

^3^School of Chemistry and Chemical Engineering, Sun Yat-sen University, Guangzhou 510275, P. R. China

^4^Chemistry and Chemical Engineering Guangdong Laboratory, Shantou 515031, China

Table of Contents

[**Materials and Methods** 3](#_Toc150705690)

[Fig. S1. Structure and photophysical properties of PSs. 11](#_Toc150705691)

[Fig. S2. Supporting data for the reaction of KI and RB. 12](#_Toc150705692)

[Fig. S3. Reaction of KI and RB with a periodically switched (on-off) 100 mT MF. 13](#_Toc150705693)

[Fig. S4. Supporting data for Fig. 2c. 14](#_Toc150705694)

[Fig. S5. MFE on the reaction of ^1^O_2_ and KI using Ce6 as PS. 15](#_Toc150705695)

[Fig. S6. Simulated MARY curve. 16](#_Toc150705696)

[Fig. S7. Photograph and illustration of the experimental setup for reaction of ^1^O_2_ and anthracene derivatives. 17](#_Toc150705697)

[Fig. S8. Supporting spectra data for MFE on the reaction of ^1^O_2_ and SOSG. 20](#_Toc150705698)

[Fig. S9. Supporting spectra data for MFE on the reaction of ^1^O_2_ and An. 22](#_Toc150705699)

[Fig. S10. Supporting spectra data for MFE on the reaction of ^1^O_2_ and ADPA. 24](#_Toc150705700)

[Fig. S11. Supporting spectra data for MFE on the reaction of ^1^O_2_ and CHO. 25](#_Toc150705701)

[Fig. S12. Supporting spectra data for MFE on the reaction of ^1^O_2_ and fatty acids. 26](#_Toc150705702)

[Fig. S13. Supporting data for MFE on the reaction of ^1^O_2_ and C11BDP in solution. 27](#_Toc150705703)

[Fig. S14. Supporting data for MFE on the reaction of ^1^O_2_ and C11BDP in GUVs. 28](#_Toc150705704)

[Fig. S15. Supporting data for MFE on the reaction of ^1^O_2_ and C11BDP in HeLa cells. 29](#_Toc150705705)

[Fig. S16. Alterations of oxidative lipidomics. 30](#_Toc150705706)

[Fig. S17. Supporting data for MFE on the ^1^O_2_ induced cytotoxicity. 31](#_Toc150705707)

[Fig. S18. Cytotoxicity of RB without photo-irradiation in different applied MF (0 and 250 mT) against HeLa cells. 32](#_Toc150705708)

[Fig. S19. MFE on the cytotoxicity using Ce6 as PS. 33](#_Toc150705709)

[Fig. S20. Dependence of Δ*mfe*(-) values on *IC*_50,0_ against different cell lines. 34](#_Toc150705710)

[Fig. S21. Unprocessed western blots and quantification results for Fig. 4d and 4e. 35](#_Toc150705711)

[Fig. S22. Supporting data for MFE on *in vivo* PDT efficacy. 36](#_Toc150705712)

[Fig. S23. H&E staining images of major organs. 37](#_Toc150705713)

[**References** 38](#_Toc150705714)

**Materials and Methods**

**Materials**

All reagents and solvents were obtained commercially and used without further purification. Rose Bengal (RB), oleic acid (OA), linoleic acid (LA), 1-palmitoyl-2-oleoyl-sn-glycero-3-phosphocholine (POPC), 1-palmitoyl-2-oleoyl-sn-glycero-3-phosphoglycerol (POPG), deuterium oxide (D_2_O) and deuterated chloroform (CDCl_3_) were purchased from Energy Chemical Co. Chlorin e6 (Ce6) was purchased from Macklin Inc. (Shanghai). Cholesterol (CHO) was purchased from Sinopharm Chemical Reagent Co., Ltd. Singlet Oxygen Sensor Green^®^ (SOSG) and C11-BODIPY^581/591^ (C11BDP) was purchased from ThermoFisher Scientific (China) Co. Potassium iodide was purchased from Beijing Chemical Industry Group Co. Cell Counting Kit-8 (CCK-8), crystal violet, 2′,7′-dichlorofluorescein diacetate (H2DCFDA) and Annexin V-FITC/PI apoptosis assay kit were purchased from Beyotime Biotechnology Co.

**Antibodies**

β-Actin (8H10D10) Mouse mAb (#3700), cleaved PARP (Asp214) (D64E10) XP^®^ Rabbit mAb (#5625), Caspase-3 antibody (#9662), cleaved Caspase-3 (Asp175) antibody (#9661), Bax antibody (#2774), Bcl-2 (124) mouse mAb (#15071), anti-mouse IgG HRP-linked antibody (#7076) and Anti-rabbit IgG HRP-linked Antibody (#7074) were purchased from Cell Signaling Technology (CST).

**Mice**

Five-week-old female BALB/c Nude mice were housed under standard environmental conditions. *In vivo* experiments were performed in strict accordance with the guidelines for the welfare and use of animals in cancer research [1] and was approved by the Institutional Animal Care and Use Committee of Sino research (China, Beijing) and Biotechnology Co., Ltd.

**Singlet oxygen (^1^O_2_) quantum yield (*Φ*_Δ_) and lifetime (*τ*_Δ_) detection**

The phosphorescence of photosensitizer-generated ^1^O_2_ at 1270 nm was determined in air-saturated D_2_O solutions of RB (10 μM) on an Edinburgh Analytical Instruments FLS980 lifetime and steady-state spectrometer equipped with a NIR-PMT R5509-73 detector. The solution was excited by 561 nm laser light. The quantum yield in the presence of different MFs (*Φ*_Δ, B_) was calculated using the following equation:

*Φ*_Δ,B_ = *Φ*_Δ,0_×(*I*_B_/*I*_0_) (2)

where *I* stands for the integrated area of the emission peak. Thus the MFE on *Φ*_Δ_ (*mfe*_Δ_) was identified as *mfe_Φ_* = (*Φ*_Δ,B_ − *Φ*_Δ,0_) / *Φ*_Δ,0_ × 100% = (*I*_B_ − *I*_0_) / *I*_0_ × 100%. Similarly, the MFE on the lifetime of ^1^O_2_ (*τ*_Δ_) was determined using *mfe_τ_* = (*τ*_Δ,B_ − *τ*_Δ,0_) / *τ*_Δ,0_ × 100%.

**Reaction of ^1^O_2_ and potassium iodide (KI) in aqueous solution**

KI reacts with ^1^O_2_ to afford a daughter product characterized by absorption bands centered at 300 and 350 nm. [2] The UV-Vis absorption spectra were obtained on an Agilent 8453 UV/Vis spectrometer equipped with an Agilent 89090A thermostat (± 0.1 °C). The photosensitizer (RB or Ce6) and KI were dissolved in water. The solution was exposed to LED light (561 and 635 nm for RB and Ce6, respectively; 5 mW cm^−2^), and the absorbance at 350 nm were recorded every 15 s for 5 min. The reaction rate is proportional to the slope of the absorbance at 350 nm as a function of time. External magnetic fields were supplied by an East Changing (Beijing) EM4 electromagnet. Magnetic field strengths were measured using a TUNKIA (Changsha) TD8620 magnetometer, and light power density was measured using a radiometer.

**Reaction of ^1^O_2_ and SOSG in aqueous solution**

The fluorescent probe SOSG reacts with ^1^O_2_ to afford a daughter product characterized by an intense green fluorescence emission (excited at 488 nm). The fluorescence spectra were obtained on a Horiba fluorescence spectrometer equipped with a PMT R928 detector. The photosensitizer (RB) and SOSG were dissolved in water, both to a concentration of 10 μM. The solution was exposed to 561 nm laser light (5 mW cm^−2^), and fluorescence spectra at different time points (0, 15, 30, 45, 60, 90, 120, 180 s) were recorded (Supplementary Fig. 1). The reaction rate is proportional to the slope of the fluorescence emission intensity at 525 nm as a function of time. External magnetic fields were supplied by an Oxford magneto-optical Cryofree® superconducting magnet system.

**Reaction of ^1^O_2_ and anthracene derivatives**

The reaction of An and ADPA with ^1^O_2_ was monitored by the quenching of fluorescence emission (excited at 375 nm) using a Horiba fluorescence spectrometer equipped with a PMT R928 detector. The photosensitizer (RB) and anthracene derivatives were dissolved in water, both to a concentration of 10 μM. The solution was exposed to 561 nm laser light (5 mW cm^−2^), and the fluorescence spectra at different time points were recorded (Supplementary Fig. 2-3). The reaction rate is proportional to the slope of the fluorescence emission intensity as a function of time. External magnetic fields were supplied by an Oxford magneto-optical Cryofree^®^ superconducting magnet system.

**Reaction of ^1^O_2_ and lipids**

0.2 mL of the photosensitizer (Ce6) solution (1.25 mM) was mixed with 0.2 mL of lipid solution (lipid = CHO, OA or LA; 0.25 M) in CHCl_3_-*d*. The solution was then irradiated with a 635 nm LED lamp for 1 h (20 mW cm^−2^) with different applied MFs (0, 15, 250, 800 mT) while cooled with water-packs. Conversions were determined by ^1^H NMR spectroscopy using 1,1,2,2-tetrabromoethane as an internal standard on the basis of the starting amount of lipid. External magnetic fields were supplied by an Oxford magneto-optical Cryofree® superconducting magnet system.

**Reaction of ^1^O_2_ and C11BDP in EtOH**

The reaction was monitored by fluorometry using a Horiba fluorescence spectrometer. Fluorescence of C11BDP was measured by simultaneous acquisition of the green (488/520 nm) and red signals (532/590 nm). The photosensitizer (Ce6) and C11BDP (1 mM in DMSO) were dissolved in water, both to a concentration of 20 μM. The solution was exposed to 635 nm light (20 mW cm^−2^) under different MFs, and the fluorescence spectra at different time points were recorded. The MFE on the oxidation state of C11BDP in different conditions was assessed using the intensity ratio oxidized- C11BDP/original-C11BDP (*R* = *I*_Green_/*I*_Red_): *mfe* = (*R*_B_ − *R*_0_)/*R*_0_ × 100%. External magnetic fields were supplied by an Oxford magneto-optical Cryofree^®^ superconducting magnet system.

**Preparation of vesicles**

GUVs were made by a gentle hydration method [3]. POPC/POPG/CHO (20:2.2:2.0 mg) were dissolved in 10 mL of a chloroform/methanol mixture (v/v = 3:1) in a glass round-bottom flask. The solvent was evaporated slowly using a rotary evaporator. The round-bottom flask was left inside a vacuum chamber overnight to remove residual solvent. The lipid film was prehydrated by keeping it under a stream of water-saturated nitrogen for 30 min. About 10 mL of GUV buffer (PBS containing 200 mM sucrose, pH 7.4) was then gently transferred into the tube. The round-bottom flask was left inside the 37 °C water bath overnight. C11BDP solution (1 mM in DMSO) was added to the solution of GUVs to a final concentration of 20 μM C11BDP, and the mixture was stirred gently for 30 min. To get confocal images, the vesicles were placed in a PBS solution (pH 7.4) containing 200 mM glucose.

**Cell culture**

HeLa cells were used and cultured in Dulbecco’s modified Eagle medium (DMEM, Corning) supplemented with 10% fetal bovine serum (FBS), 1% penicillin and streptomycin. Cells were grown at 37 °C in a humidified atmosphere containing 5% CO_2_. The external magnetic field for *in vitro* experiments was supplied by an Oxford magneto-optical Cryofree^®^ superconducting magnet system.

**Reaction of ^1^O_2_ and C11BDP in GUVs and living cells**

The reaction was monitored by a Nikon A1R-si laser scanning confocal microscope. The oxidation level was determined from the changes in the green (λ_ex_: 488 nm; λ_em_: 515/30 nm) and red (λ_ex_: 543 nm; λ_em_: 595/30 nm) signals. Ce6 was dissolved in water or incubated into HeLa cells (12 h) to a final concentration of 20 μM in both cases. The suspension or cells was exposed to 635 nm light (5 mW cm^−2^) for 10 min under different MFs, and the confocal images were captured. 3 GUVs or 20 cells /group were randomly selected for the intensity quantification, MFE presented as mean ± SD.

**Targeted oxidative lipidomics**

**Chemicals and agents.** All eicosanoids and deuterated internal standards were purchased from Cayman Chemical. HPLC-grade acetonitrile (ACN) and methanol (MeOH) were purchased from Merck (Darmstadt, Germany). MilliQ water (Millipore, Bradford, USA) was used in all experiments. Acetic acid was purchased from Sigma-Aldrich. CNW Poly-Sery MAX SPE cartridges were from ANPEL Co. (Shanghai, PRC). The stock solutions of standards were prepared at the concentration of 5 μg/mL in MeOH. All stock solutions were stored at -80°C. The stock solutions were diluted with MeOH to working solutions before analysis. (Guangzhou IGE Biotechnology Ltd. provides technology assistant.)

**Sample preparation.** Caki-1 cells (2 × 10^5^) were seeded and cultured in 10 cm plates for 24 h, and were treated with PBS (Group A) or RB (10 µM, Groups B-E). 12 h later, the cells were washed 3 times with PBS and kept under photo-irradiation (400−700 nm white light, 5 mW cm^−2^) for 10 min, with or without a MF (0, 0, 15, 250, and 800 mT for Group A-E respectively). After photoirradiation, cells were washed with PBS and collected by centrifugation (950*g*, 5 min). The sample stored at -80 °C refrigerator was thawed on ice. Cell consolidation and separation: 200uL PHS buffer was taken from the first tube and vortexed evenly, transferred to the second tube until the fifth tube, and then 150uL PHS was added into the first tube. In the same operation, the buffer was merged into the fifth tube and vortexed evenly, and divided into 6 equal parts of 50uL each, one of which was used for cell protein determination. Divide 50 μL cell suspension and add 200 μL MeOH/ACN (v/v=1:1,) solution containing internal standard and vortexed for 5 min. The sample was placed in liquid nitrogen for 5 min and on the dry ice for 5 min, and then thawed on ice and vortexed for 2 min. This freeze-thaw circle was repeated three times in total. The protein was precipitated at low temperature (-20 °C) for 30 min. The sample was centrifuged at 12000 rpm for 10 min (4 °C). The all supernatant was collected and transferred. Repeat the extraction once and combine the supernatants. The eicosanoids in supernatants were extracted using Poly-Sery MAX SPE columns (ANPEL). Prior to analysis, the eluent was dried under vacuum and redissolved in 100 μL of MeOH/water (v/v=1:1) for UPLC/MS/MS analysis. 50 μL cell suspension was divided, frozen and thawed for 3 times, centrifuged at 12,000 r/min for 10 min, and the supernatant was taken to determine the protein concentration by BCA Protein Assay kit. 3 parallel samples were prepared for Group A and B and 5 for Group C-E to allow a statistical analysis for *mfe*.

**HPLC and ESI-MS/MS conditions.** The sample extracts were analyzed using an LC-ESI-MS/MS system (UPLC, ExionLC AD; MS, QTRAP^®^ 6500+ System). The analytical conditions were as follows, HPLC: column, Waters ACQUITY UPLC HSS T3 C18 (100 mm×2.1 mm i.d., 1.8 µm); solvent system: water with 0.04% acetic acid (A), ACN with 0.04% acetic acid (B); The gradient was 0-2.0 min from 0.1% to 30%B; 2.0-4.0 min to 50% B; 4.0-5.5 min to 99% B, which was maintained for 1.5 min; and 6.0-7.0 min reduced to 0.1% B and maintained for 3.0 min. flow rate, 0.4 mL/min; temperature, 40 °C; injection volume: 10 μL. Linear ion trap (LIT) and triple quadrupole (QQQ) scans were acquired on a triple quadrupole-linear ion trap mass spectrometer (QTRAP), QTRAP® 6500+ LC-MS/MS System, equipped with an ESI Turbo Ion-Spray interface, operating in negative ion mode and controlled by Analyst 1.6.3 software (Sciex). The ESI source operation parameters were as follows: ion source, ESI-; source temperature 550 ℃; ion spray voltage (IS) -4500 V; curtain gas (CUR) was set at 35 psi, respectively. Eicosanoids were analyzed using scheduled multiple reaction monitoring (MRM). Data acquisitions were performed using Analyst 1.6.3 software (Sciex). Multiquant 3.0.3 software (Sciex) was used to quantify all metabolites. Mass spectrometer parameters including the declustering potentials (DP) and collision energies (CE) for individual MRM transitions were done with further DP and CE optimization. A specific set of MRM transitions were monitored for each period according to the metabolites eluted within this period [4-7].

**Data processing.** Unsupervised PCA (principal component analysis) was performed by statistics function prcomp within R. The data was unit variance scaled before unsupervised PCA. Identified metabolites were annotated using KEGG compound database, annotated metabolites were then mapped to KEGG Pathway database. Pathways with significantly regulated metabolites mapped to were then fed into MSEA (metabolite sets enrichment analysis), their significance was determined by hypergeometric test’s P-Values.

**Cell viability assay**

Cells (786-O, Caki-1, CT26, HeLa, B16, HemECs, U937, MCF-7, A549, and HepG2; 2 × 10^3^) were seeded in 96-well plates, cultured for 24 h, and treated with RB or PBS. 12 h later, the cells were washed 3 times with PBS. For dark cytotoxicity, the cells were kept with or without a MF in the dark for the designed time. After incubation in DMEM (with FBS) in the dark for another 24 h, 10 µL of CCK-8 solution and 90 µL DMEM were added into each well and a follow-up incubation was performed for 30 min. The absorbance at 450 nm was measured using a 96-well plate reader. The viability of cells was calculated using the following equation:

CV = (*A*_s_ – *A*_b_) / (*A*_c_ – *A*_b_) × 100% (3)

where CV = cell viability, *A*_s_, *A*_c_ and *A*_b_ represent the absorbance of cells incubated with photosensitizer, cell control (no photosensitizer), and blank control (wells containing neither cells nor the studied photosensitizer), respectively.

For photocytotoxicity, the cells were treated following the dark cytotoxicity protocol outlined above. After washing out the photosensitizer or PBS, the cells were photo-irradiated in a MF (0-800 mT) for the designated time and incident power, which were optimized as 10 min and 5 mW cm^−2^ respectively. 400-700 nm white light and 635 nm red light (LED) were used for RB and Ce6 (PS) respectively. Each measurement was performed 3 times to allow a statistical analysis for *mfe*.

**Flow cytometry analysis for apoptosis**

HeLa cells (2 × 10^4^) were seeded and cultured in 6-well plates for 24 h, and were treated with PBS (Group A) or RB (30 µM, Group B-E). 12 h later, the cells were washed 3 times with PBS and kept with or without a MF (0, 0, 15, 250, and 800 mT for Group A-E respectively) under photo-irradiation (400−700 nm white light, 5 mW cm^−2^) for 10 min. After incubation for another 24 h, the cells were stained using the Annexin V-FITC/PI apoptosis kit and quantified by flow cytometry. Each measurement was performed 3 times to allow a statistical analysis.

**Western blot analysis**

HeLa cells (2 × 10^4^) were seeded and cultured in 10 cm plates for 24 h, and were treated following the same procedure as in the flow cytometry analysis. 24 h after exposure to a MF (0, 15, 250, or 800 mT; with photoirradiation), cells were washed with PBS and collected by centrifugation (950*g*, 5 min). Proteins were extracted from the cells using RIPA Lysis Buffer (Middle). Target proteins were detected with primary antibodies recognizing β-Actin, Caspase-3, cleaved Caspase-3, Bax, Bcl-2 and cleaved PARP respectively. Images were acquired with a Bio-Rad ChemiDoc Touch imaging system. Quantification for the MFE on the level of Bax/Bcl-2 ratio, active Cas-3 and cleaved PARP was carried out by calculating the integrated density (*D*) using Image J, data were normalized to β-Actin. *mfe* = (*D*_B_ – *D*_0_) / *D*_0_ × 100%. Each measurement was performed 3 times to allow a statistical analysis for *mfe*. Representative original blots are shown in Fig. S21.

***In vivo* therapy**

*In vivo* experiments were performed in strict accordance with the guidelines for the welfare and use of animals in cancer research [1] and was approved by the Institutional Animal Care and Use Committee of Sino research (China, Beijing) and Biotechnology Co., Ltd. Five-week-old female BALB/c Nude mice were housed under standard environmental conditions. All animal procedures were approved by the Institutional Animal Care and Use Committee of Beijing University of Agriculture. A syngeneic model was established by subcutaneously inoculating 1×10^6^ HeLa cells (in 200 µL PBS) into the right flank of each mouse. Mice were divided into 9 groups: (1) PBS-dark, (2) PBS (no MF), (3) PBS-15 mT, (4) PBS-250 mT, (5) PBS-800 mT, (6) RB (no MF), (7) RB-15 mT, (8) RB-250 mT, and (9) RB-800 mT. Each group consisted of 9 mice. RB (1.0 mg kg^−1^) or an equal volume of PBS were intratumorally injected. All groups except Group (1) received photo-irradiation (400-700 nm, 100 mW cm^−2^, 10 min) 5 min post-injection. The tumor regions of mice in all Groups except (1), (2) and (6) were additionally exposed to the designated MF using an electromagnet during photo-irradiation. The tumor sizes were monitored in the following 14 days with a digital caliper every other day and calculated as volume = (length) × (width)^2^ / 2. 3 mice in each group were sacrificed on the 14^th^ day post-injection, and the tumor tissues were collected for photographing and weighing.

**Histology**

Mice were sacrificed on the 14^th^ day post-injection. Tumor tissues and main organs (heart, liver, spleen, lung and kidney) were collected for histology. The harvested tissues were fixed with 10% neutral formalin solution. A Hematoxylin and Eosin (H&E) stain was conducted to analyze the toxicity toward tumor and normal organs (Supplementary Fig. 5).

**Data analyses**

Theoretical simulation was performed with Wolfram Mathematica 13.1. Microscopy and Western blot images were analyzed and quantified with the ImageJ software. Flow cytometry data were analyzed using Flow Jo 10. Data were presented as mean ± SD. Unless otherwise stated, experiments were performed 3 times for *mfe* calculation (n = 3). Student’s t-test was used to assess differences between means, and denoted by **p* < 0.05, ***p* < 0.01 and ****p* < 0.005. *p* < 0.05 was considered significant. n.s. = no significance.


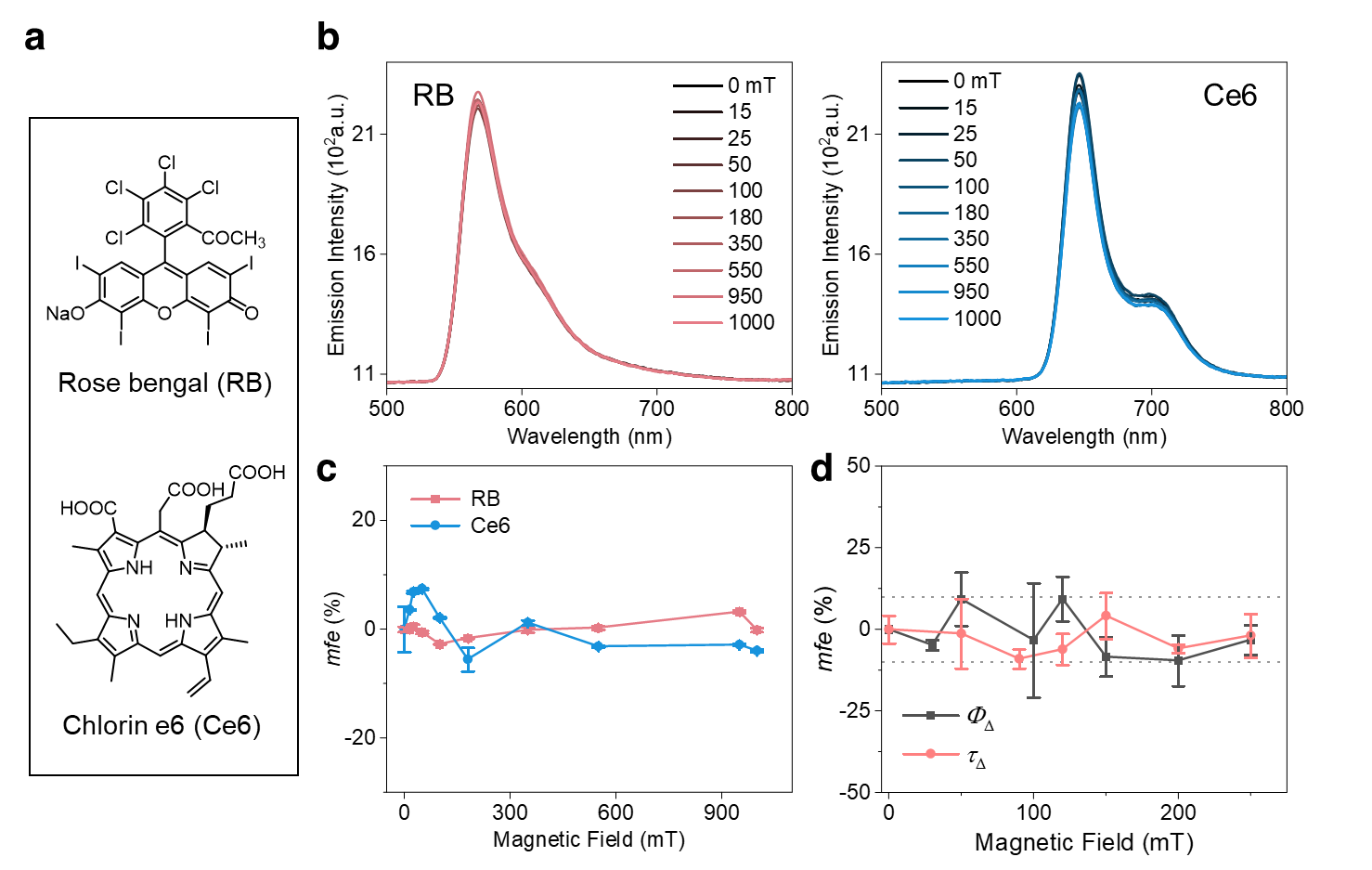


Fig. S1. Structure and photophysical properties of PSs.

(a) Structures of PSs RB and Ce6. (b) Emission spectra of RB (left) and Ce6 (right) under different MFs in the range of 0-1000 mT. (c) Magnetic field dependence of luminescence intensity of PSs. No obvious variation was found in under the MF strengths in a range of 0-1000 mT. (d) MARY spectra on the ^1^O_2_ quantum yield (*Φ*_Δ_) and lifetime (*τ*_Δ_) using RB as PS.


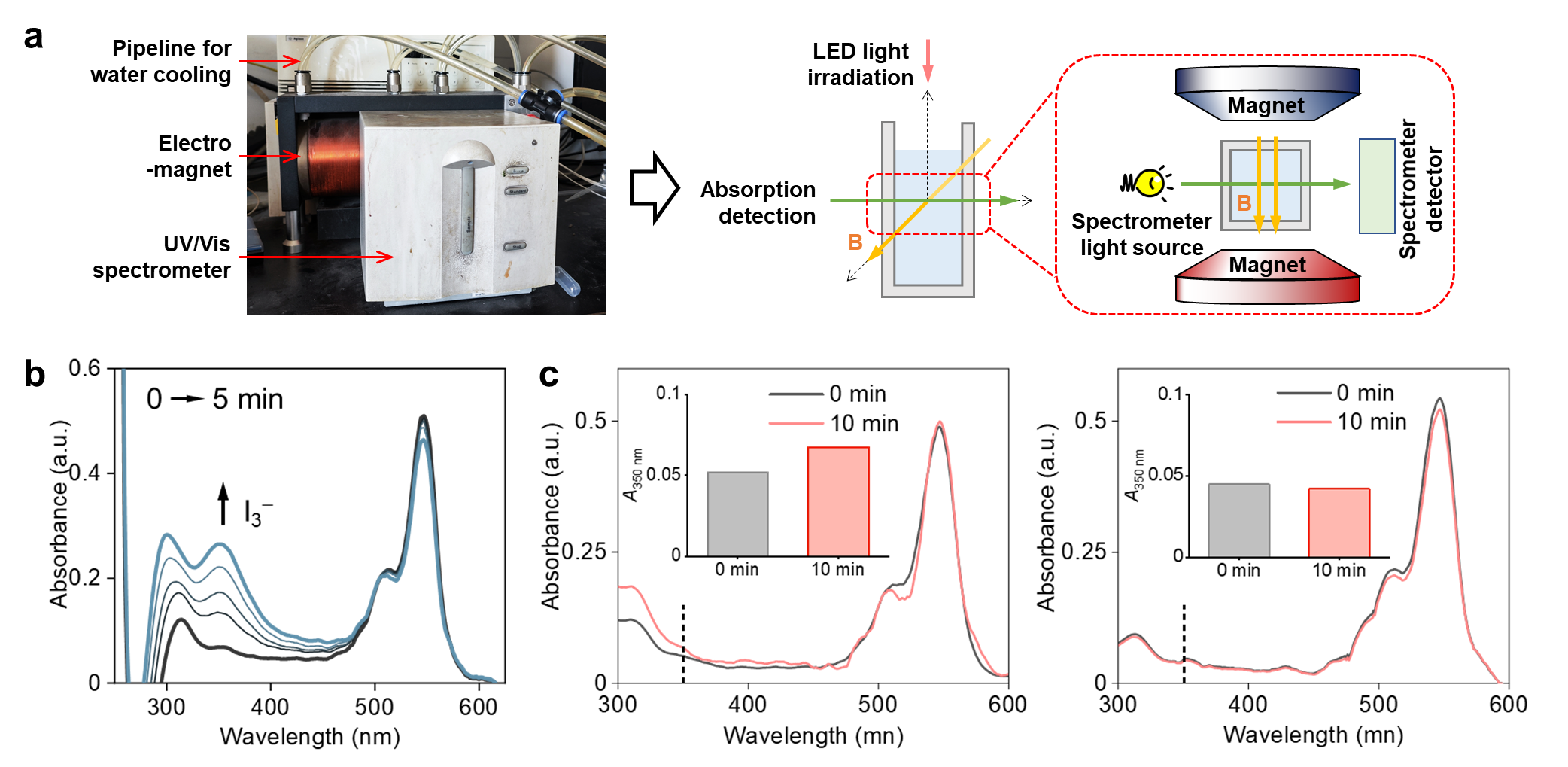


Fig. S2. Supporting data for the reaction of KI and RB.

(a) Photograph and illustration of the experimental setup for reaction in an external magnetic field. (b-c) UV-Vis absorption spectra of KI and RB in H_2_O recorded as a function of time: (b) under irradiation and (c) control experiments without irradiation (left) or irradiated under N_2_ atmosphere (right). Insets presents the absorbance changes at 350 nm, showing the negligible generation of I_3_^−^. RB: 10 μM; KI: 10 mM; irradiated at 561 nm, 5 mW cm^−2^.


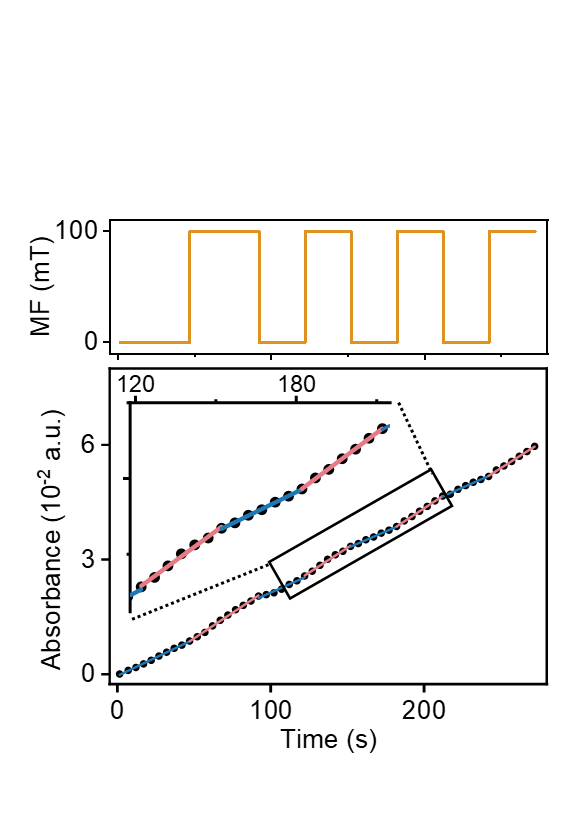


Fig. S3. Absorbance changes at 350 nm of the irradiated solution with a periodically switched (on-off) 100 mT MF.

RB: 10 μM; KI: 10 mM; irradiated at 561 nm, 5 mW cm^−2^.


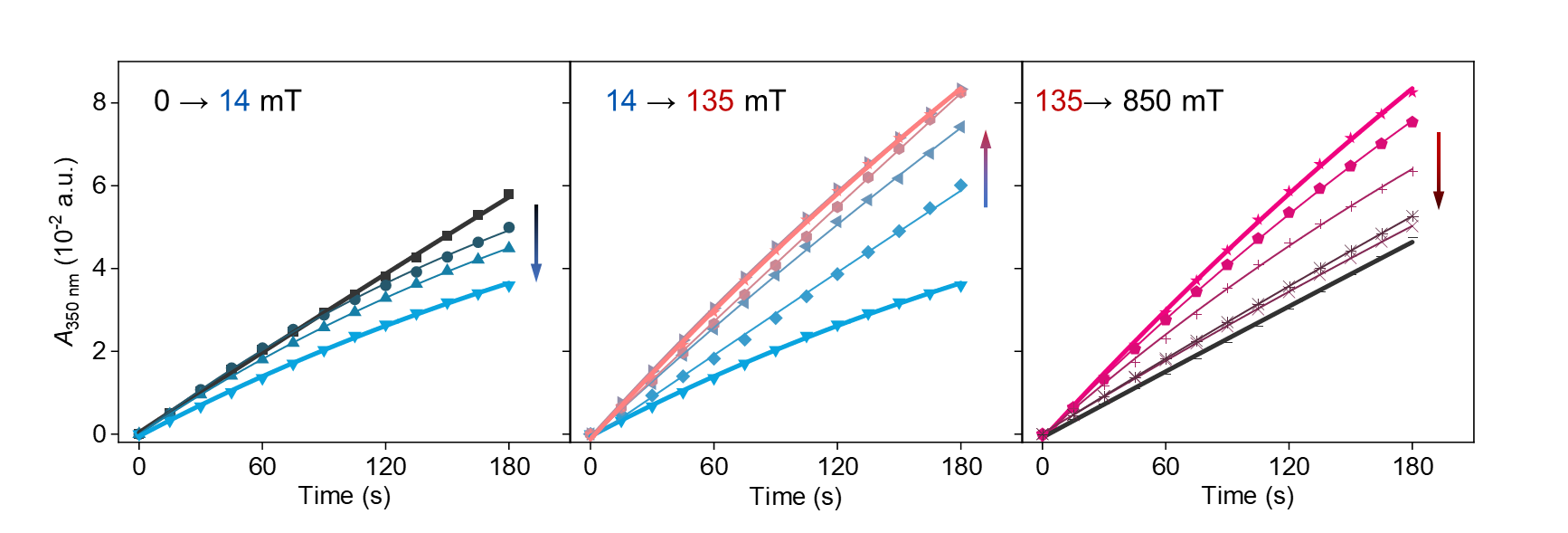


Fig. S4. Supporting data for Fig. 2c.

Absorbance changes at 350 nm of the irradiated I^−^ and RB solution under air atmosphere under different MFs (0-800 mT). RB: 10 μM; KI: 10 mM; irradiated at 561 nm, 5 mW cm^−2^.


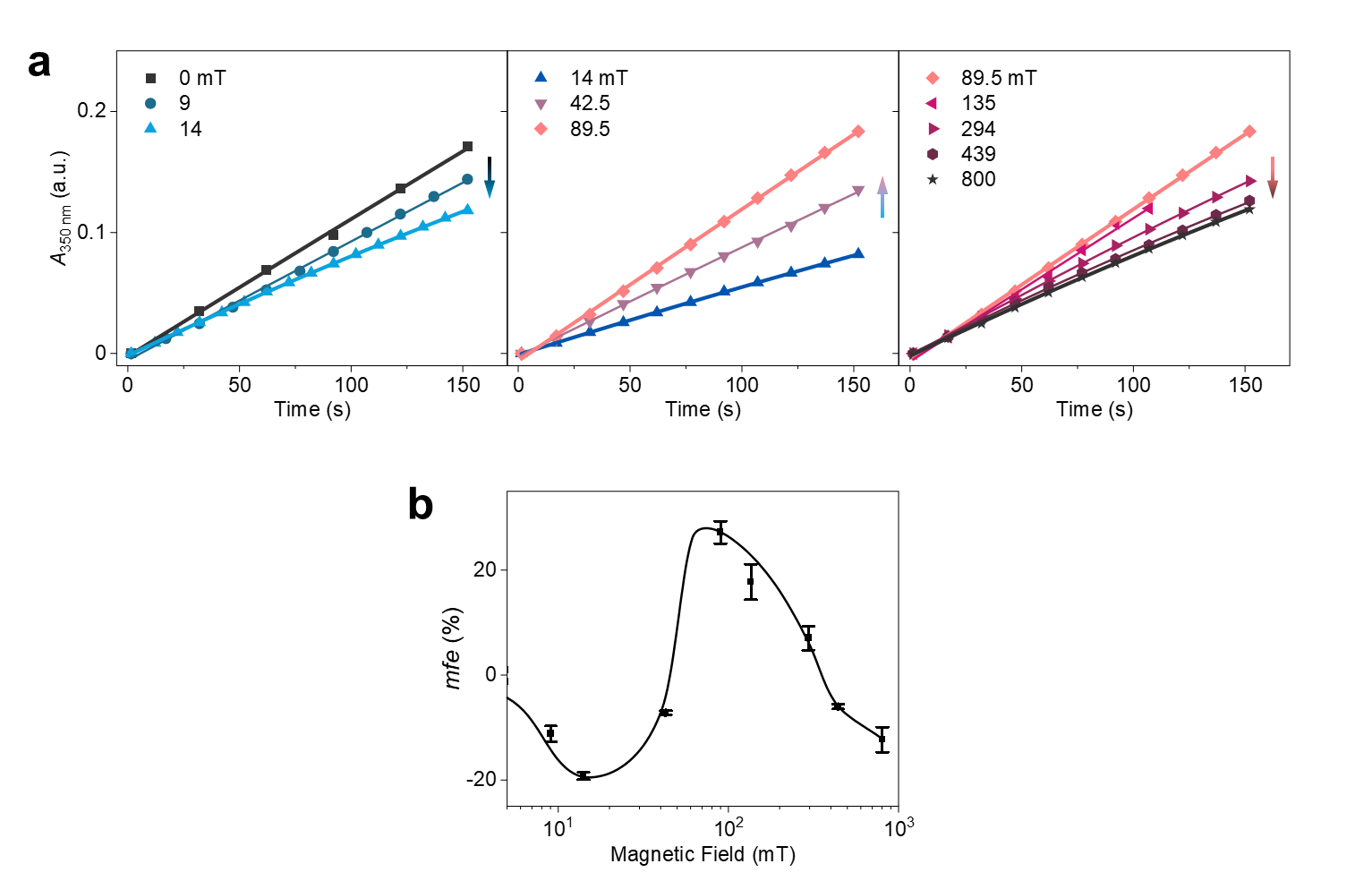


Fig. S5. MFE on the reaction of ^1^O_2_ and KI using Ce6 as PS.

(a) Absorbance changes at 350 nm of the irradiated I^−^ and Ce6 solution under air atmosphere under different MFs (0-800 mT). (b) Dependence of the *mfe* values on the MF strength for the reaction with I^−^. *mfe* = (*r*_B_ − *r*_0_)/*r*_0_ × 100%, data presented as mean ± SD (n = 3). Ce6: 10 μM; KI: 10 mM; irradiated at 635 nm, 5 mW cm^−2^.


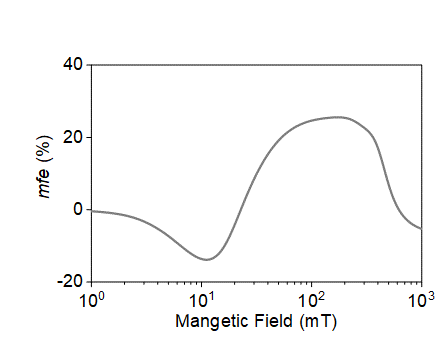


Fig. S6. Simulated MARY curve.

Using spin dynamic simulations methods developed by Hore[8]. The code is shown in Supplementary Code.


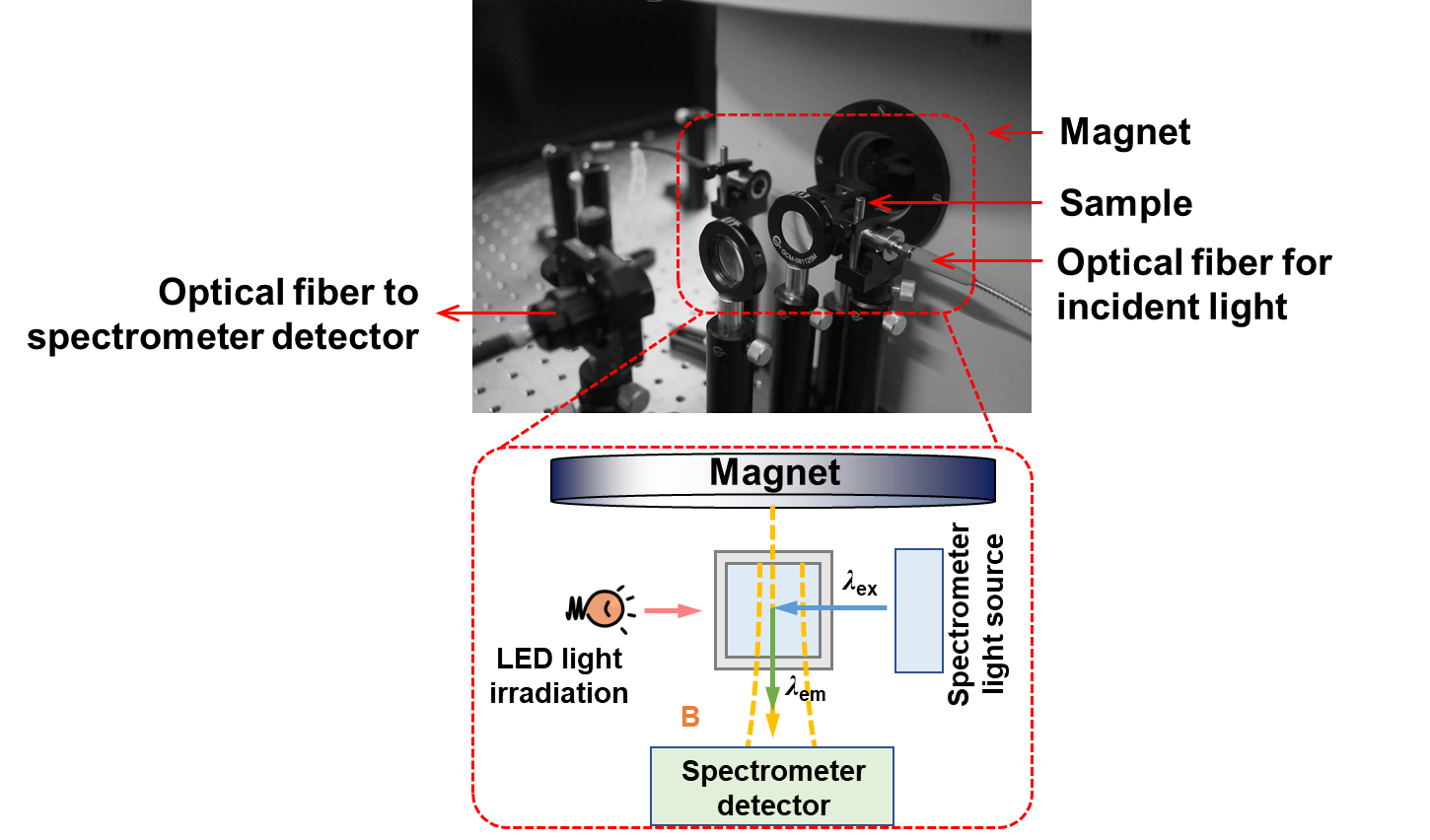


Fig. S7. Photograph and illustration of the experimental setup for reaction of ^1^O_2_ and anthracene derivatives.


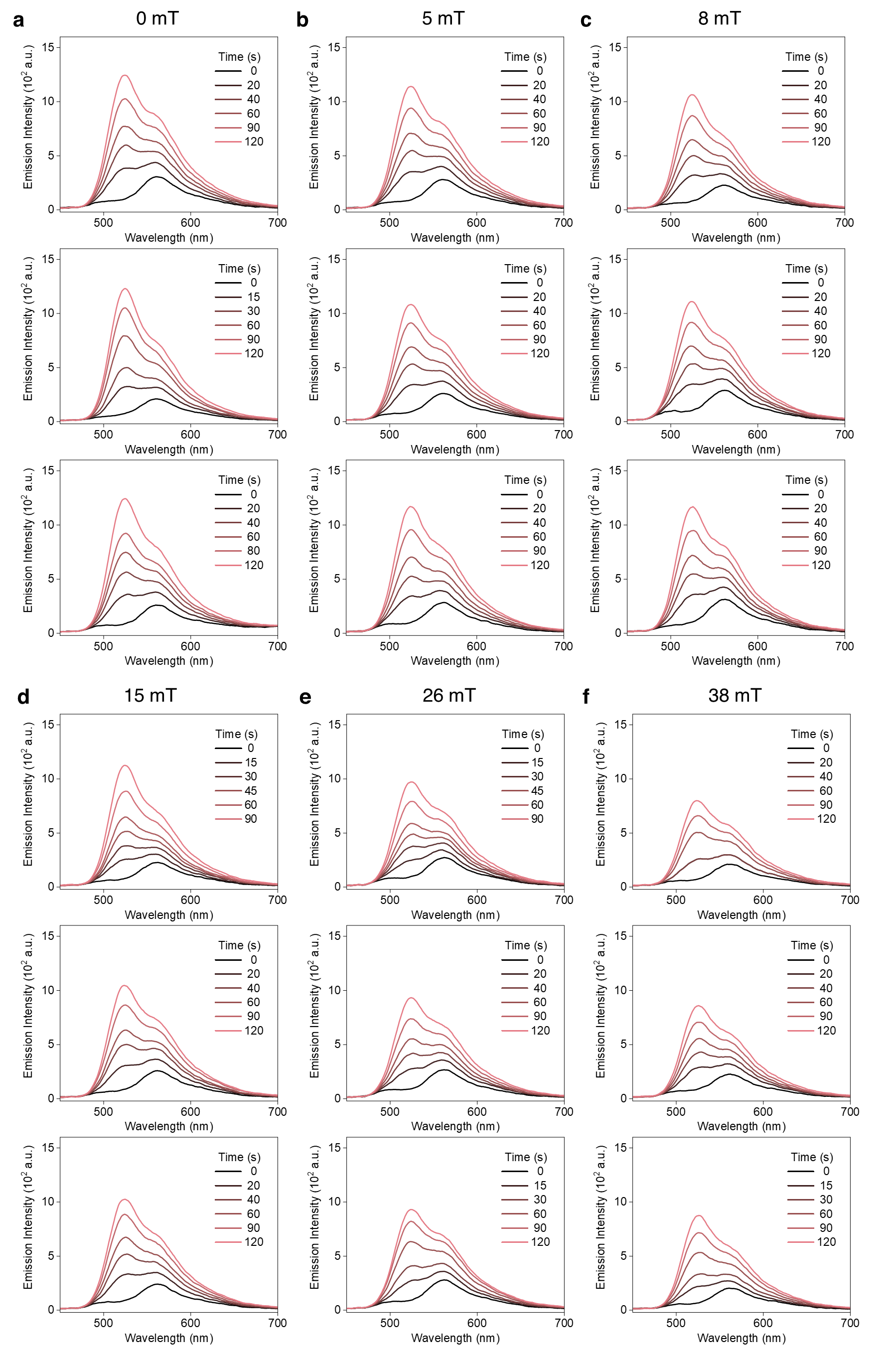


(Continued Fig. S8)


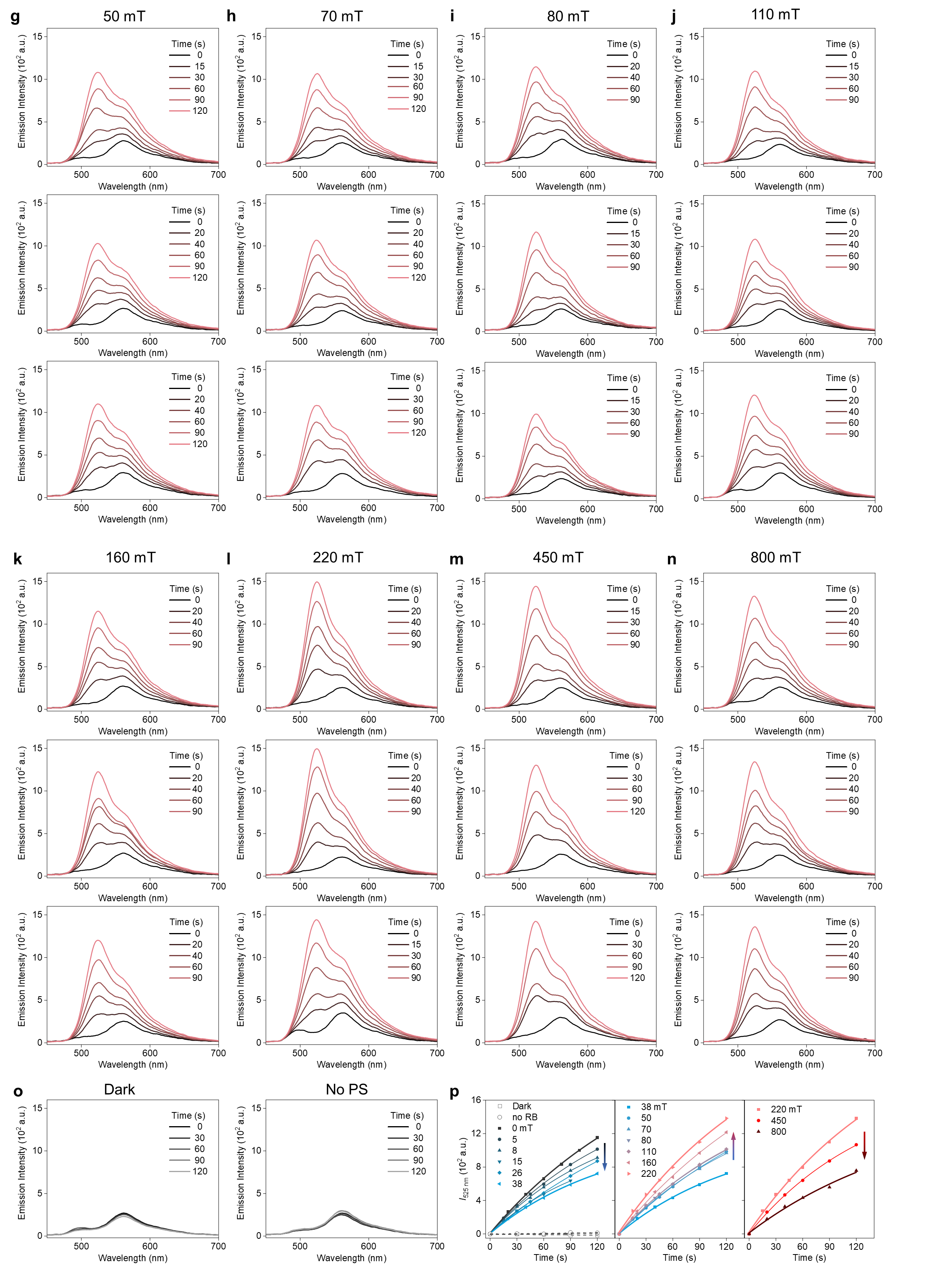


Fig. S8. Supporting spectra data for MFE on the reaction of ^1^O_2_ and SOSG.

(a)-(n): Emission spectra data of SOSG in the presence of RB under 0, 5, 8, 15, 26, 38, 50, 70, 80, 110, 160, 220, 450, 800 mT, respectively. (o) Control emission spectra recorded without irradiation (left) or RB (right) respectively. (p) Emission intensity changes (at 525 nm) recorded as function of time under the MF strengths in a range of 0-800 mT, Dashed lines present the data recorded without irradiation (gray) or irradiated in the absence of RB (dark gray). RB: 10 μM, SOSG: 10 μM; irradiated at 561 nm, 5 mW cm^−2^.


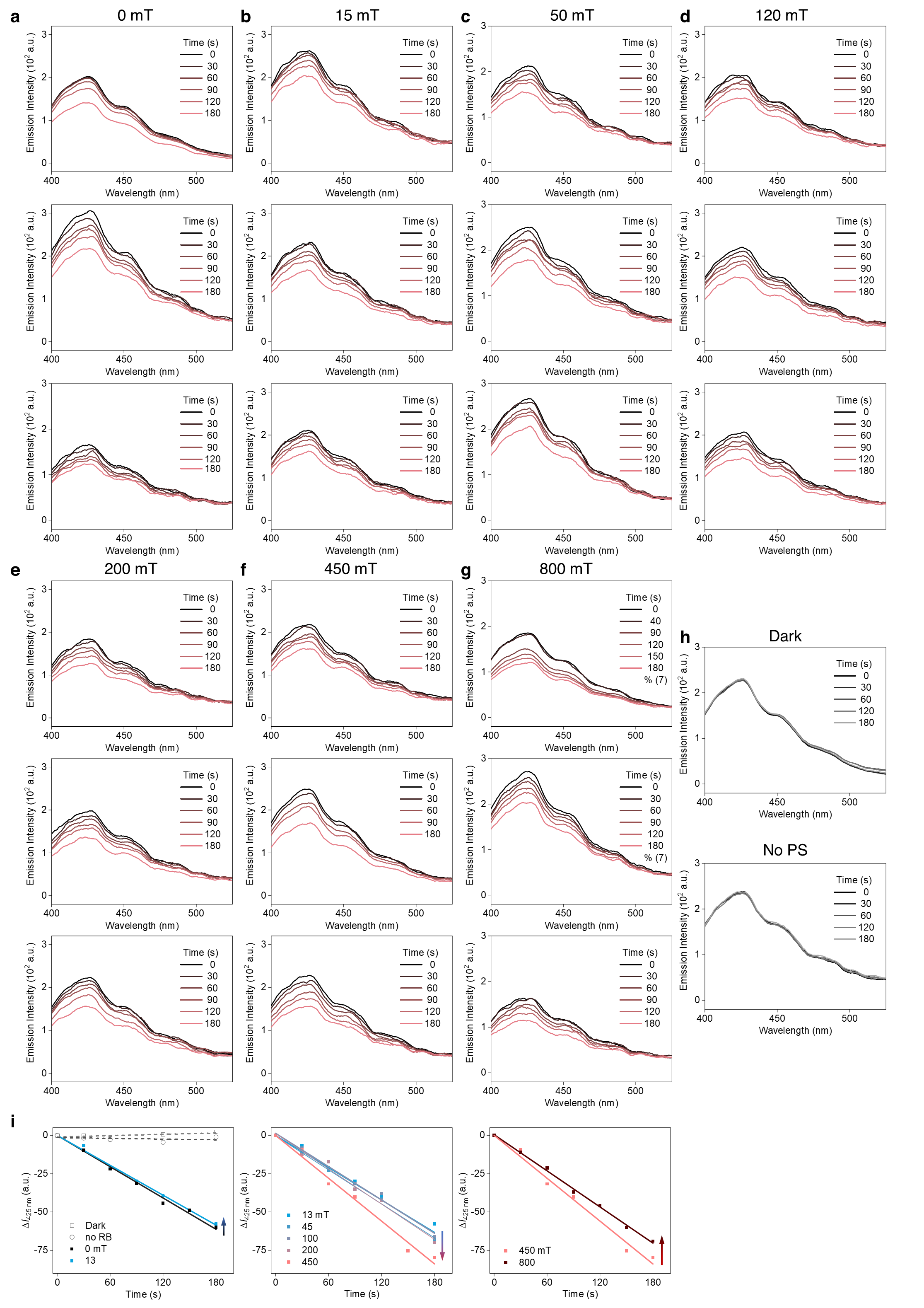


Fig. S9. Supporting spectra data for MFE on the reaction of ^1^O_2_ and An.

(a)-(g): Emission spectra data of An in the presence of RB under 0, 15, 50, 120, 200, 450, 800 mT, respectively. (h) Control emission spectra recorded without irradiation (up) or RB (down) respectively. (i) Emission intensity changes (at 425 nm) recorded as function of time under the MF strengths in a range of 0-800 mT, Dashed lines present the data recorded without irradiation (gray) or irradiated in the absence of RB (dark gray). RB: 10 μM, An: 10 μM; irradiated at 561 nm, 5 mW cm^−2^.


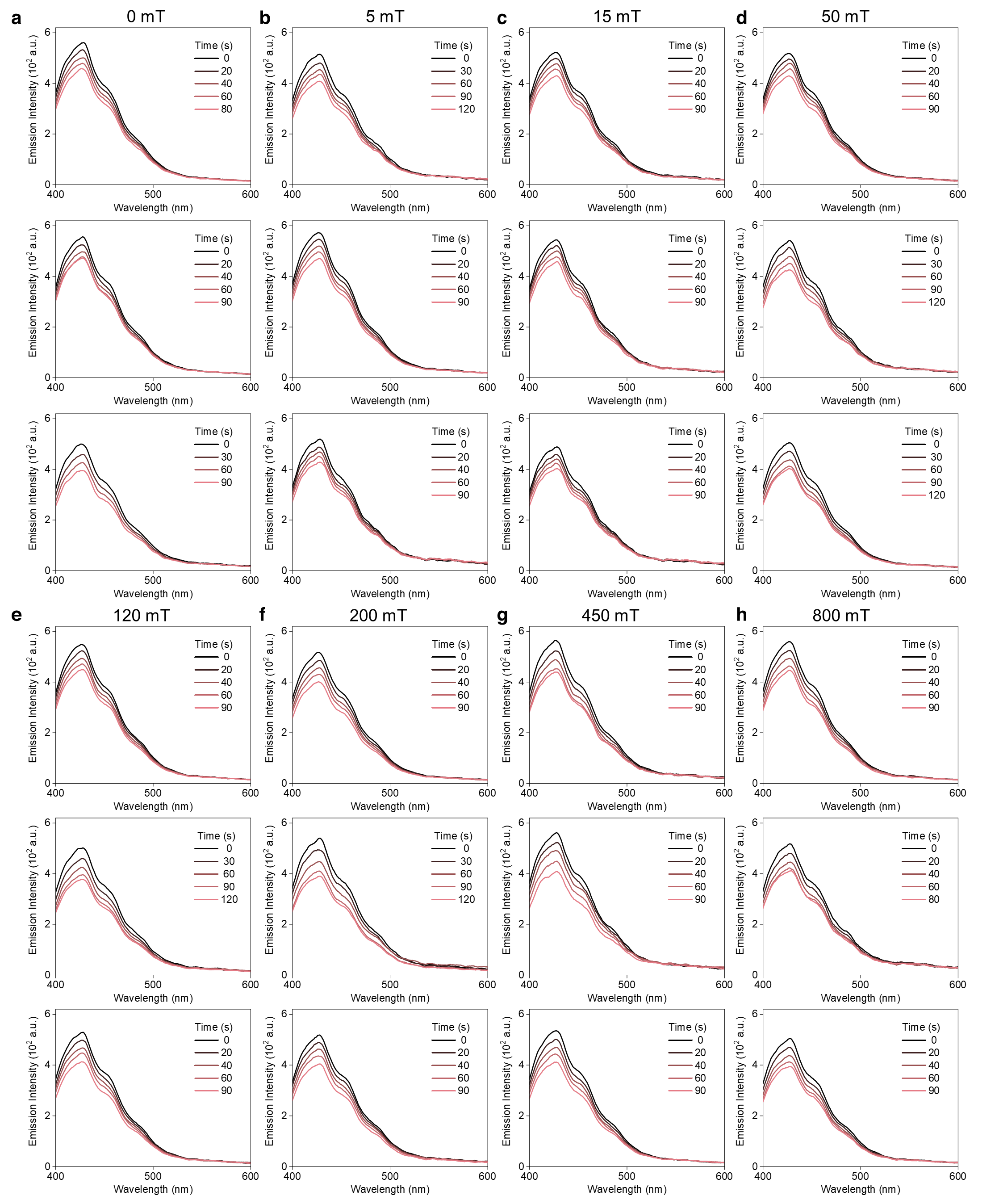


(Continued Fig. S10)


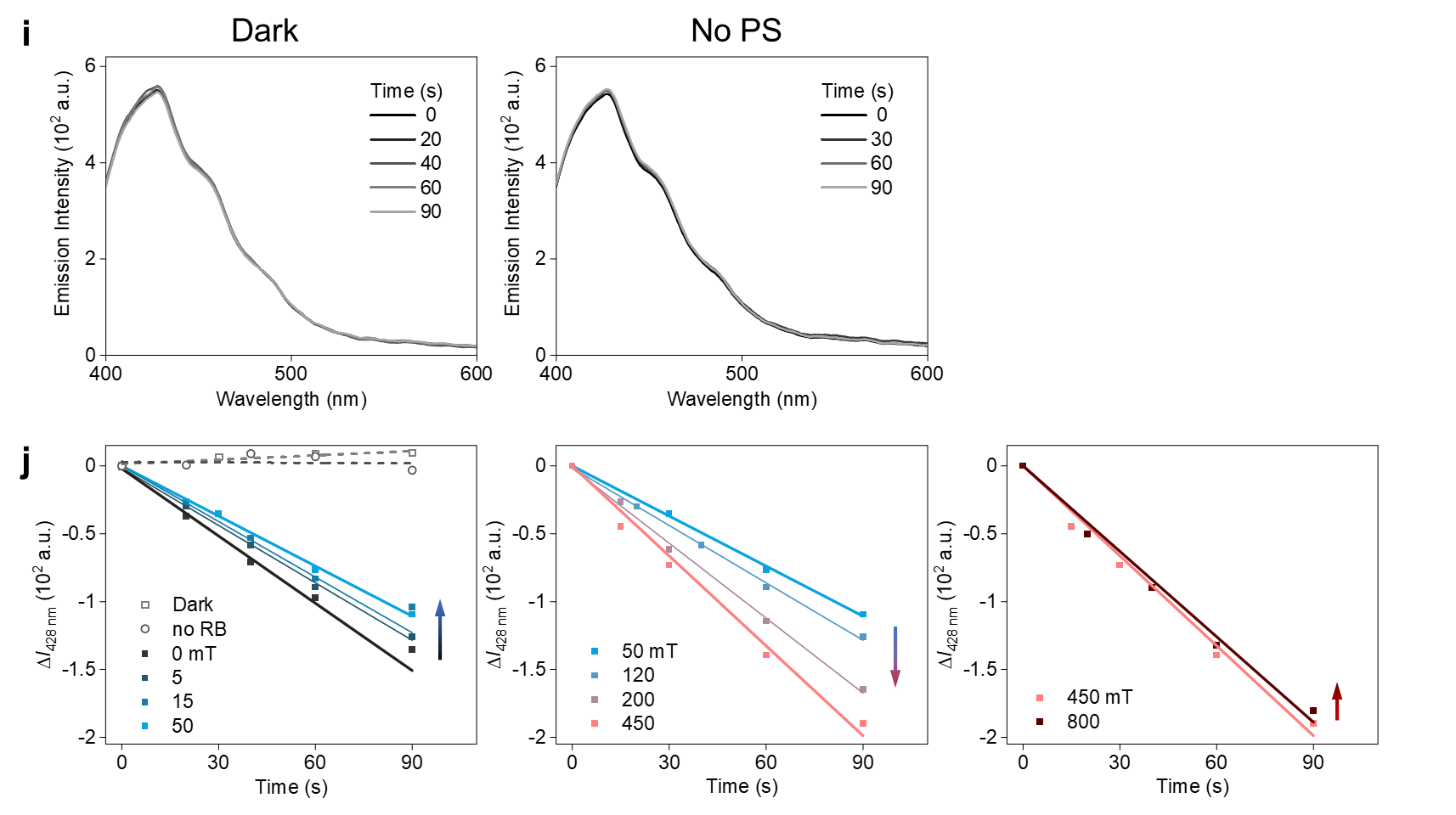


Fig. S10. Supporting spectra data for MFE on the reaction of ^1^O_2_ and ADPA.

(a)-(h): Emission spectra data of An in the presence of RB under 0, 5, 15, 50, 120, 200, 450, 800 mT, respectively. (i) Control emission spectra recorded without irradiation (left) or RB (right) respectively. (j) Emission intensity changes (at 428 nm) recorded as function of time under the MF strengths in a range of 0-800 mT, Dashed lines present the data recorded without irradiation (gray) or irradiated in the absence of RB (dark gray). RB: 10 μM, ADPA: 10 μM; irradiated at 561 nm, 5 mW cm^−2^.


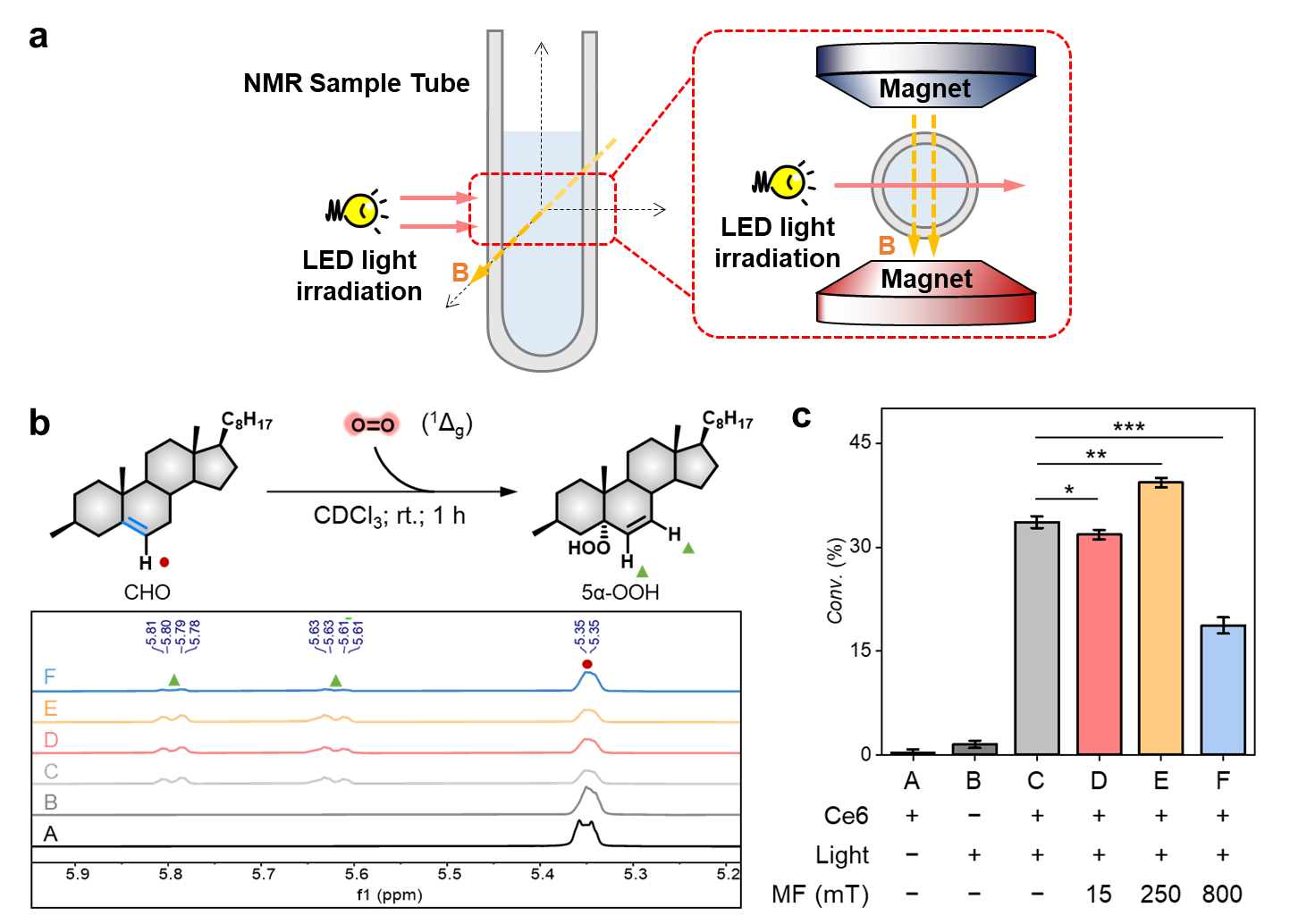


Fig. S11. Supporting spectra data for MFE on the reaction of ^1^O_2_ and CHO.

(a) Illustration of the experimental setup for the reaction in an external magnetic field. (b) The ^1^H NMR spectra of CHO oxidation reaction under different MFs in CHCl_3_-*d*: C: 0 mT, D: 15 mT, E: 250 mT, F: 800 mT. A: CHO and Ce6 system without irradiation (0 mT). B: CHO irradiated for 1h in the absence of Ce6 (0 mT). (b) Conversion (*Conv.*) of CHO under different MFs calculated from (a). CHO: 0.05 mmol, PS: Ce6 (0.5%); irradiation condition: 635 nm, 20 mW·cm^−1^, 1h, air atmosphere. Using 1,1,2,2-tetrabromoethane as internal standard for conversion calculation.


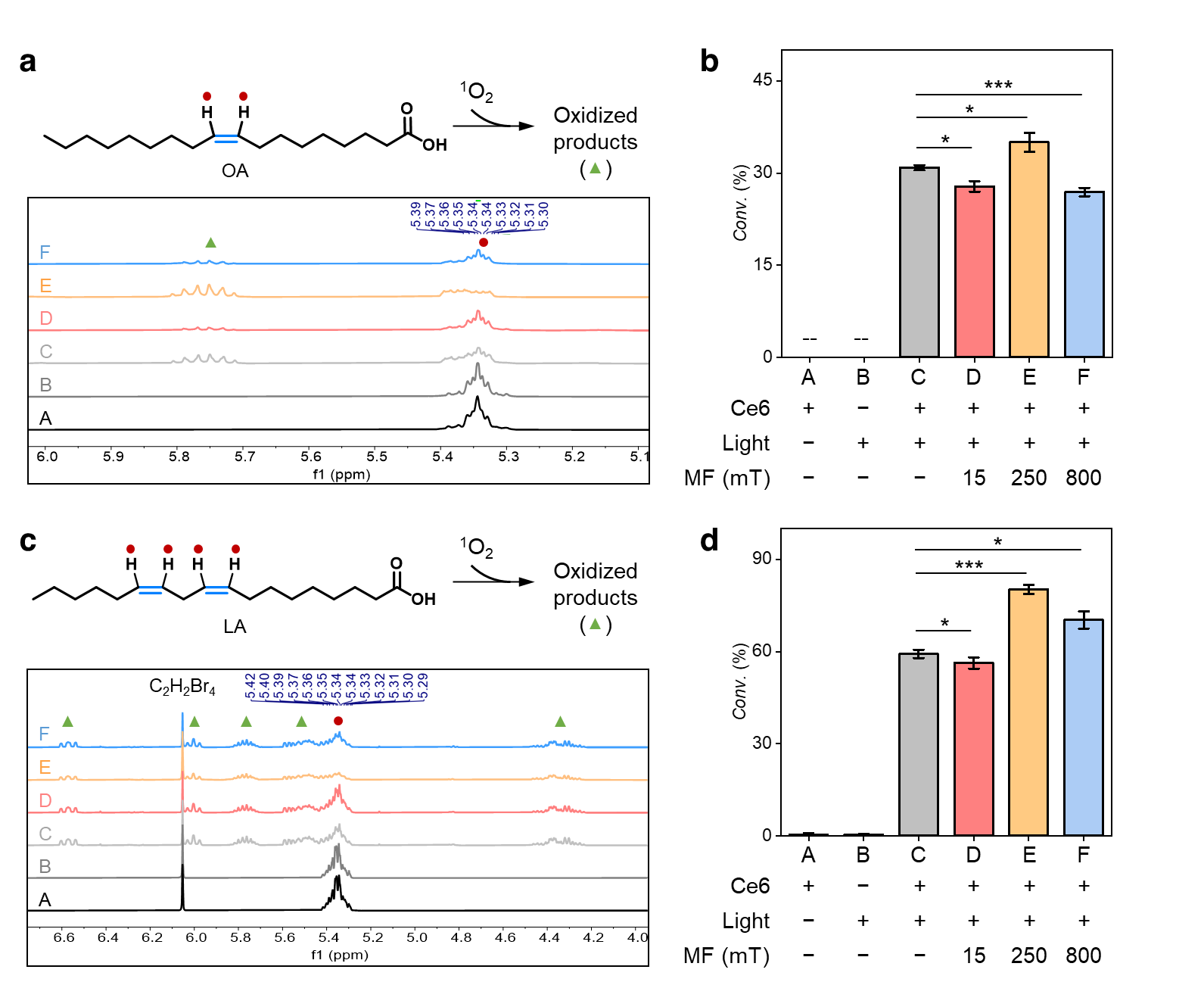


Fig. S12. Supporting spectra data for MFE on the reaction of ^1^O_2_ and fatty acids.

(a) The ^1^H NMR spectra of OA oxidation reaction under different MFs in CHCl_3_-*d*. (b) Conversion (*Conv.*) of OA under different MFs calculated from (a). (c) The ^1^H NMR spectra of LA oxidation reaction under different MFs in CHCl_3_-*d*. (d) Conversion (*Conv.*) of LA under different MFs calculated from (c). Lipids: 0.05 mmol, PS: Ce6 (0.5%); irradiation condition: 635 nm, 20 mW·cm^−1^, 1h, air atmosphere. Using 1,1,2,2-tetrabromoethane as internal standard for conversion calculation.


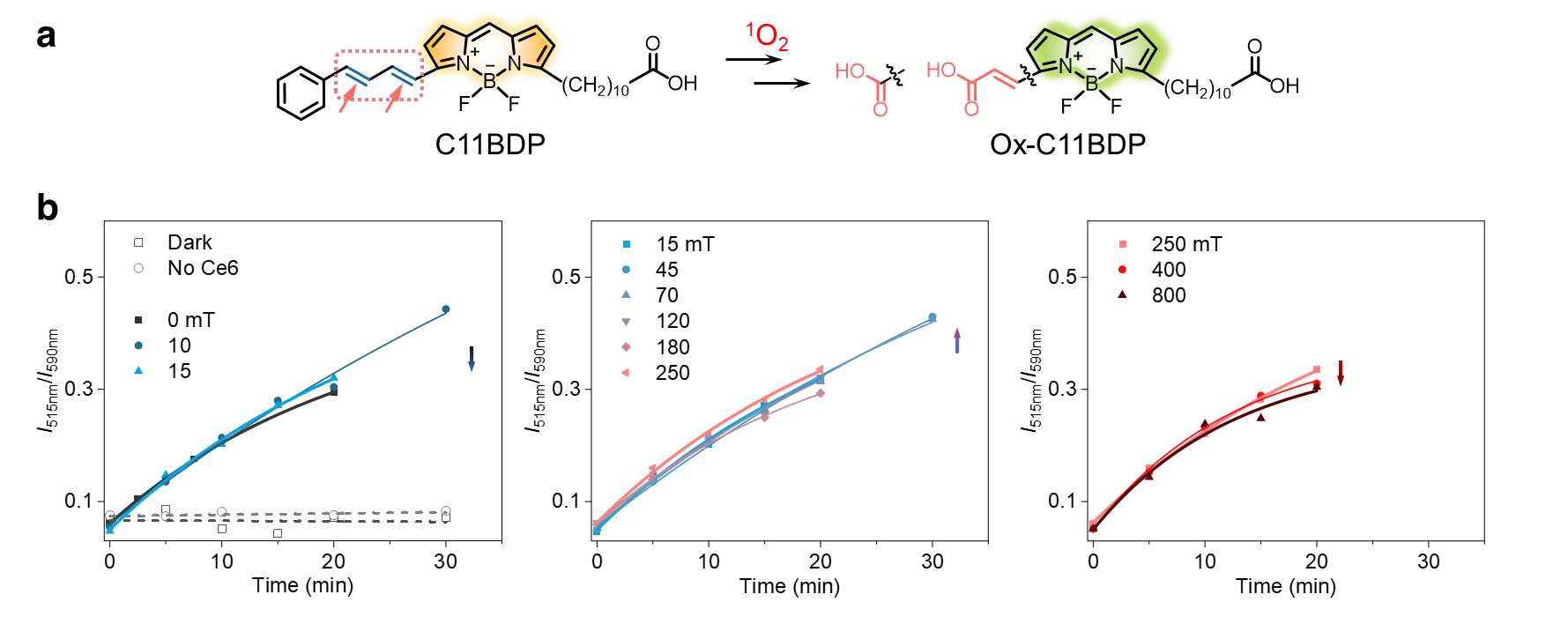


Fig. S13. Supporting data for MFE on the reaction of ^1^O_2_ and C11BDP in solution.

(a) Reaction pathway for the C11BDP oxidation. The oxidized form is recorded as Ox-C11BDP. (b) Changes of the Ox-C11BDP/C11BDP ratio (*I*_515 nm_/*I*_590 nm_) under the MF strengths in a range of 0-800 mT. Dashed lines present the data recorded without irradiation (gray) or irradiated in the absence of Ce6 (dark gray). Ce6: 20 μM, C11BDP: 20 μM. Irradiation condition: 635 nm, 20 mW cm^−2^.


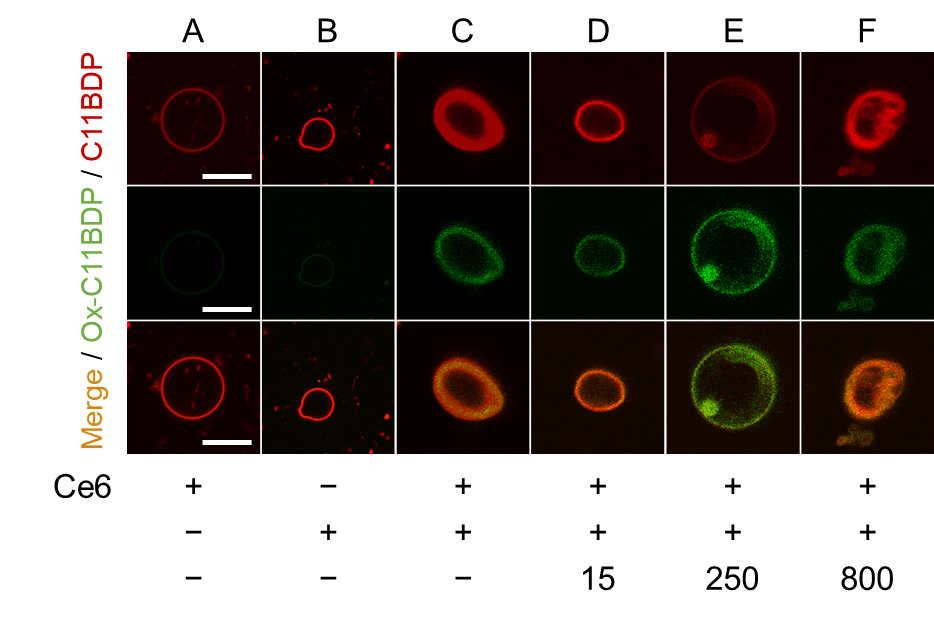


Fig. S14. Supporting data for MFE on the reaction of ^1^O_2_ and C11BDP in GUVs.

Representative fluorescence images of GUVs treated with PBS or Ce6 and stained with C11BDP, and irradiated (10 min) at different external MFs (0, 50, 250, 800 mT). The emission of C11BDP and Ox-C11BDP are recorded through red (λ_ex_: 543 nm; λ_em_: 595/30 nm) and green (λ_ex_: 488 nm; λ_em_: 515/30 nm) channels, respectively. Ce6: 20 μM, C11BDP: 20 μM. Irradiation condition: 635 nm, 5 mW cm^−2^, 10 min. **p* < 0.05, ***p* < 0.01, n.s.: no significance. Scale bar: 15 μm.


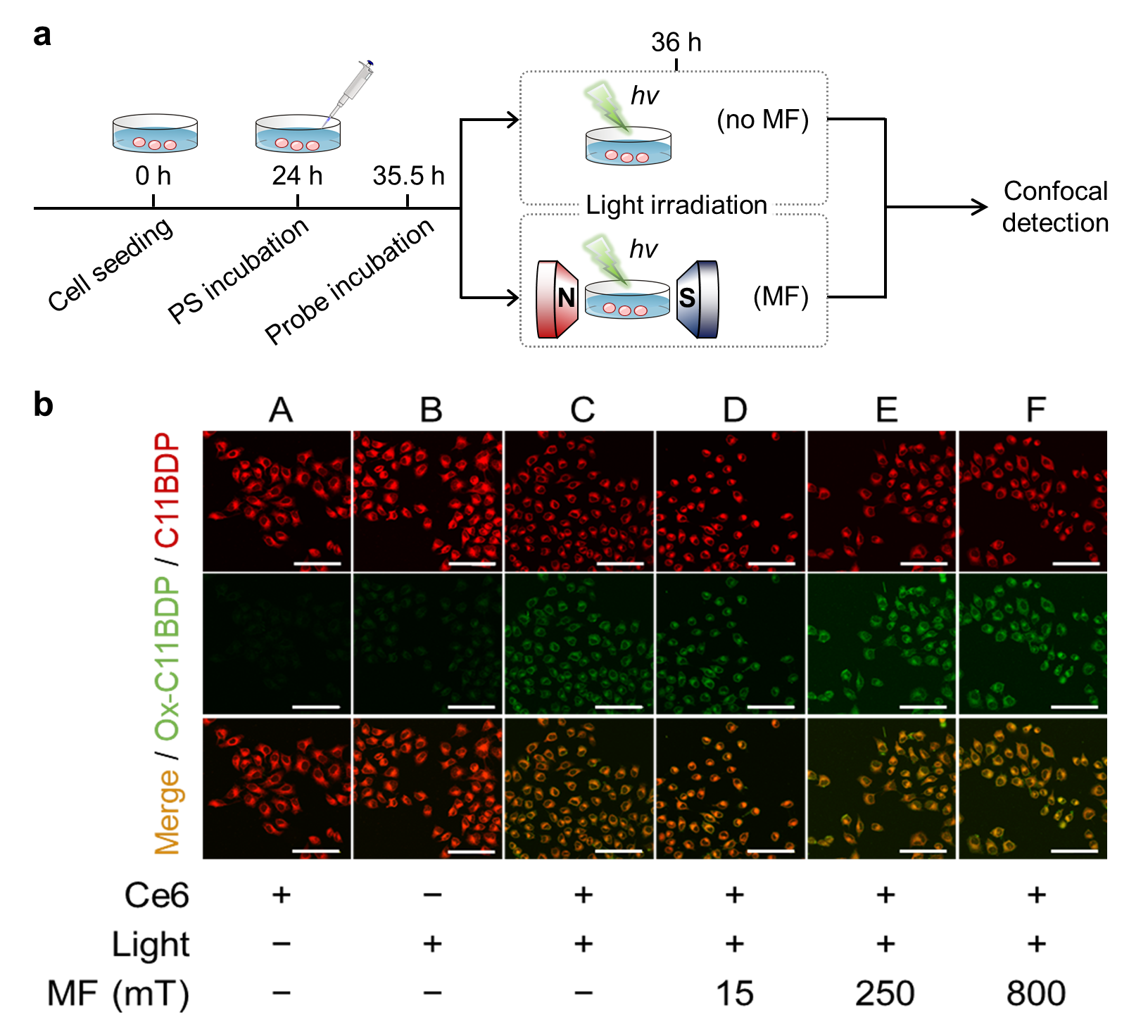


Fig. S15. Supporting data for MFE on the reaction of ^1^O_2_ and C11BDP in HeLa cells.

(a) Illustration of the experimental setup for the reaction in the presence of an external magnetic field. (b) Representative fluorescence images of HeLa cells treated with PBS or Ce6 and stained with C11BDP, and irradiated (10 min) at different external MFs (0, 50, 250, 800 mT). The emission of C11BDP and Ox-C11BDP are recorded through red (λ_ex_: 543 nm; λ_em_: 595/30 nm) and green (λ_ex_: 488 nm; λ_em_: 515/30 nm) channels, respectively. Ce6: 20 μM, C11BDP: 20 μM. Irradiation condition: 635 nm, 5 mW cm^−2^, 10 min. **p* < 0.05, ***p* < 0.01, n.s.: no significance. Scale bar: 100 nm.


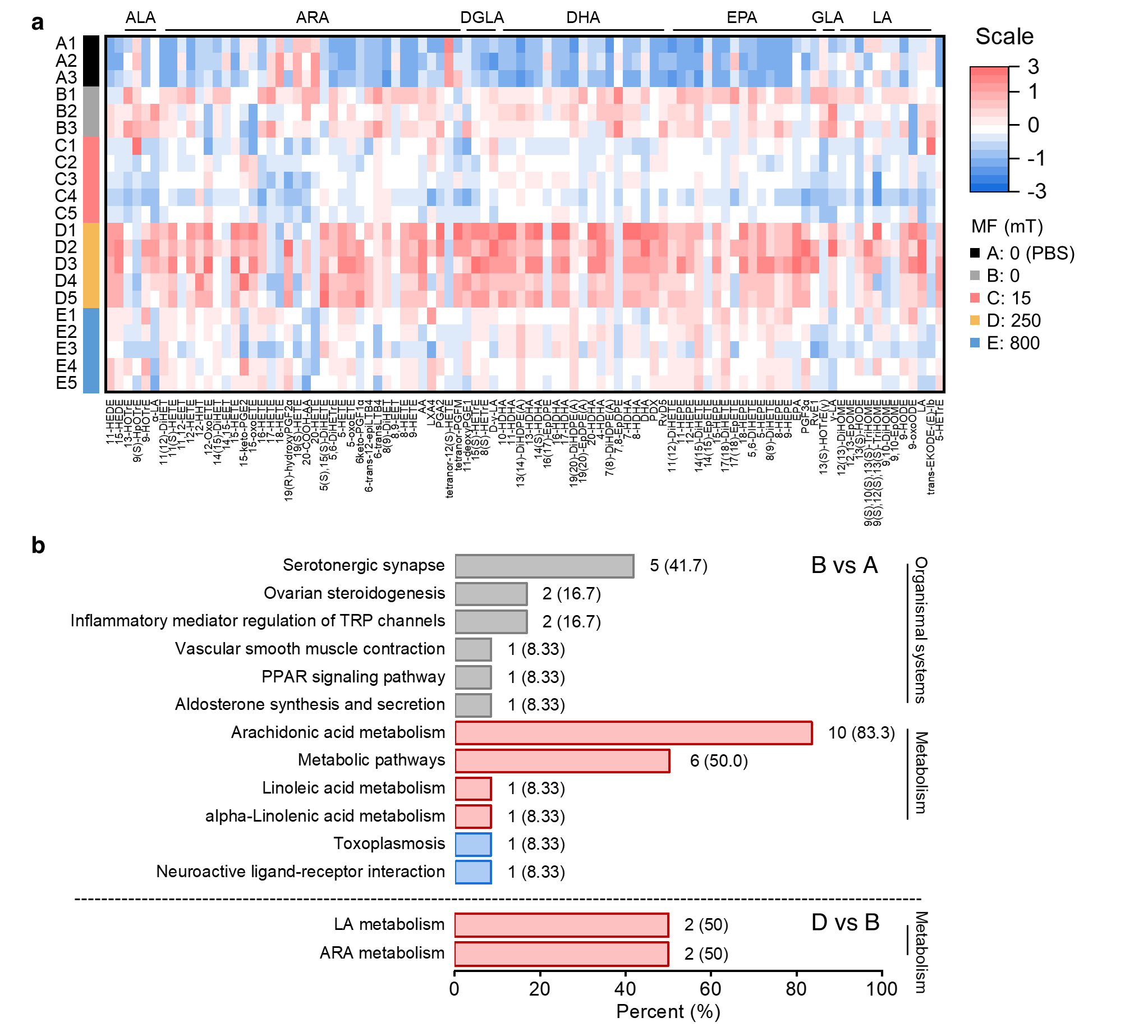


Fig. S16. Alterations of oxidative lipidomics.

(a) Heatmap of differential oxylipins between control (Group A) and PDT groups. (b) Enrichment Kyoto Encyclopedia of Genes and Genomes (KEGG) pathway analysis of ^1^O_2_ induced oxylipins (Group B vs A, top) and MFE (Group D vs B, bottom) respectively.


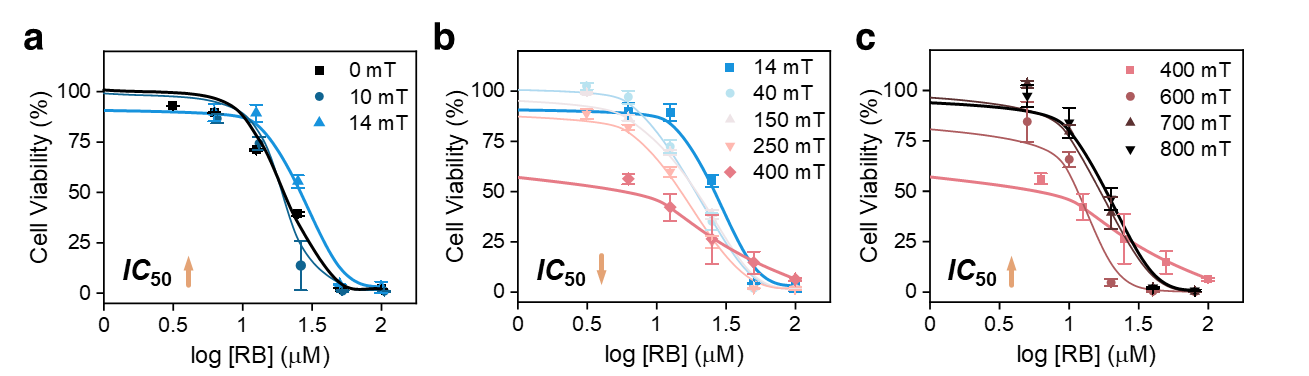


Fig. S17. Supporting data for MFE on the ^1^O_2_ induced cytotoxicity.

Photocytotoxicity of RB against HeLa cells under photo-irradiation (10 min) in applied MFs up to 800 mT, determined using a CCK-8 assay. (a) 0 and 14 mT, (b) 14−400 mT, and (c) 400−800 mT. Data presented as mean ± SD (n = 3). Irradiation condition: 400-700 nm white light, 5 mW cm^−2^, 10 min.


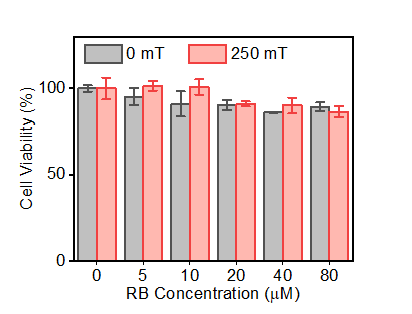


Fig. S18. Cytotoxicity of RB without photo-irradiation in different applied MF (0 and 250 mT) against HeLa cells.


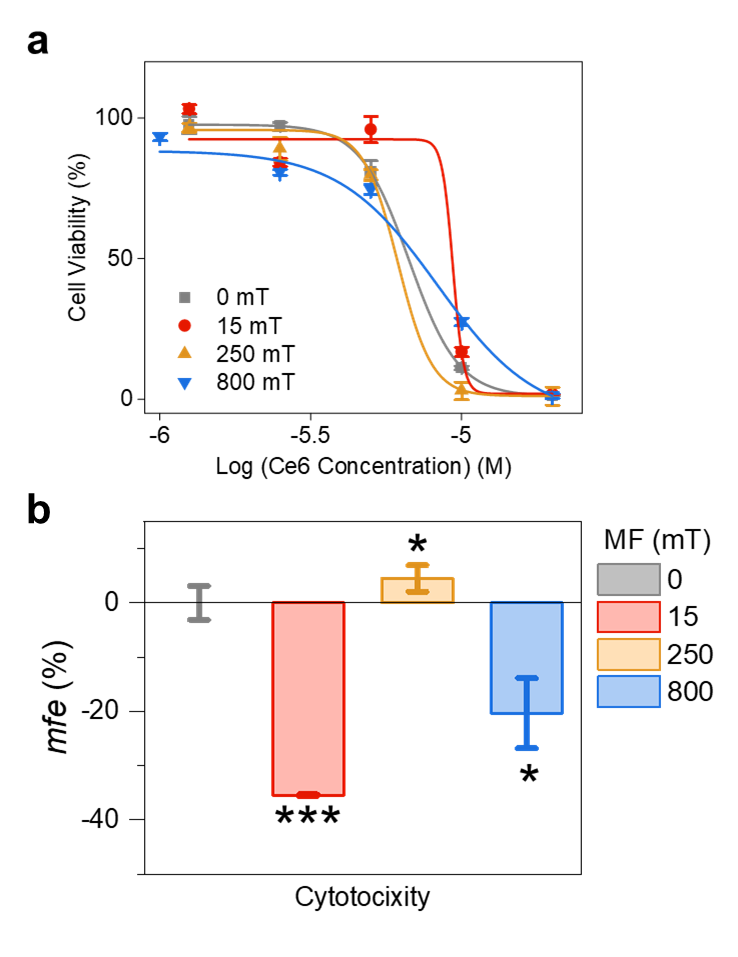


Fig. S19. MFE on the cytotoxicity using Ce6 as PS.

(a) Photocytotoxicity of Ce6 against HeLa cells under photo-irradiation (10 min) in applied MFs up to 800 mT, determined using a CCK-8 assay. (b) MFE on *IC*_50_ values. *mfe* = –(*IC*_50, B_ − *IC*_50, 0_) / *IC*_50, 0_ × 100%. Data presented as mean ± SD (n = 3). **p* < 0.05, ****p* < 0.005. Irradiation condition: 635 nm LED, 5 mW cm^−2^, 10 min.


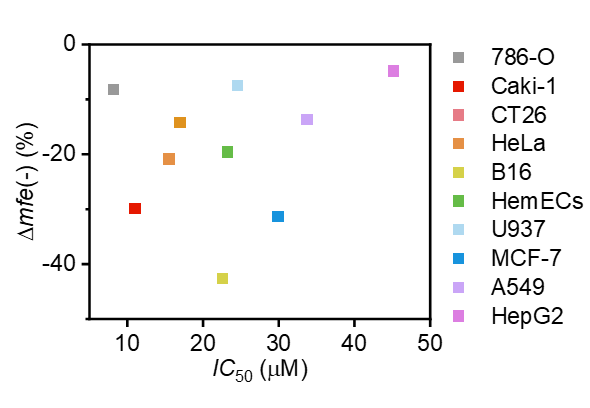


Fig. S20. Dependence of Δ*mfe(-)* values on *IC*_50,0_ against different cell lines.

RB: 20 μM. Irradiation condition: 400-700 nm white light, 5 mW cm^−2^, 10 min.


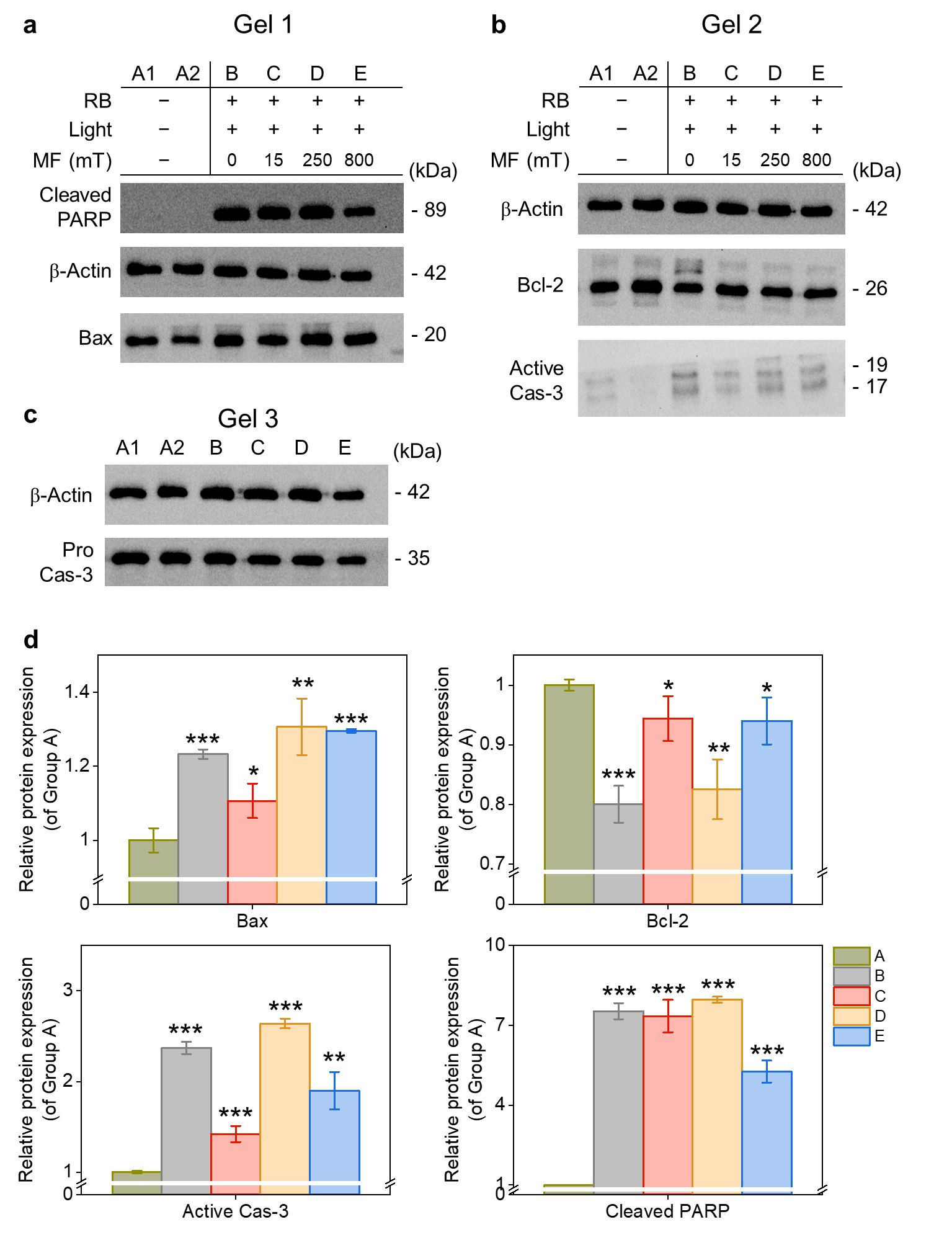


Fig. S21. Unprocessed western blots for Fig. 5d and 5e.

(a) – (c) shows cleaved PARP, Bax, active (cleaved) Cas-3, Bcl-2 and Pro Cas-3 respectively. Cells in Groups A-E were treated with PBS (A) or RB (B-E, 20 μM, 12 h) and received irradiation in 0 (A and B), 15 (C), 250 (D) and 800 mT (E) MFs. (d) Quantification results for the expression level of Bax, Bcl-2, active Cas-3 and cleaved PARP, calculated by the integrated density (*D*) in (a)-(c) using Image J. Data were normalized to β-Actin. Irradiation condition: 400-700 nm white light, 5 mW cm^−2^, 10 min.


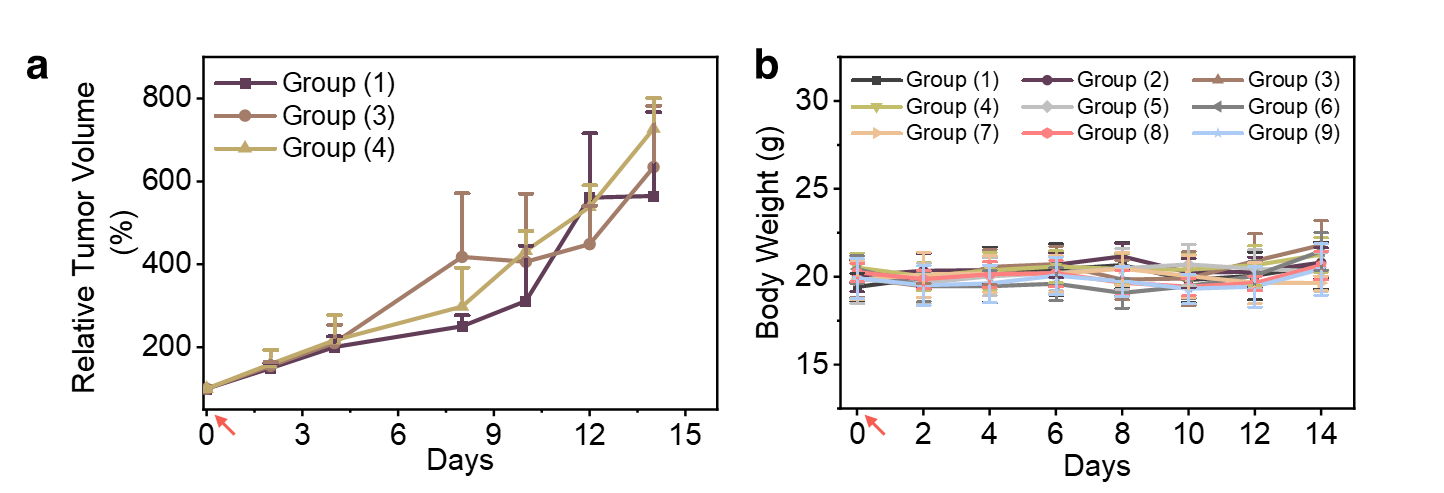


Fig. S22. Supporting data for MFE on *in vivo* PDT efficacy.

(a) Tumor growth profiles for mice groups (1): PBS-dark, (3): PBS-15 mT and (4): PBS-250 mT. (b) Body weights curves of the mice in different treatment groups. All groups received photo-irradiation 5 min post-injection (arrow in (A)), 400−700 nm white light, 100 mW cm^-2^, 10 min. Data presented as mean ± SD (n =9 mice/group).


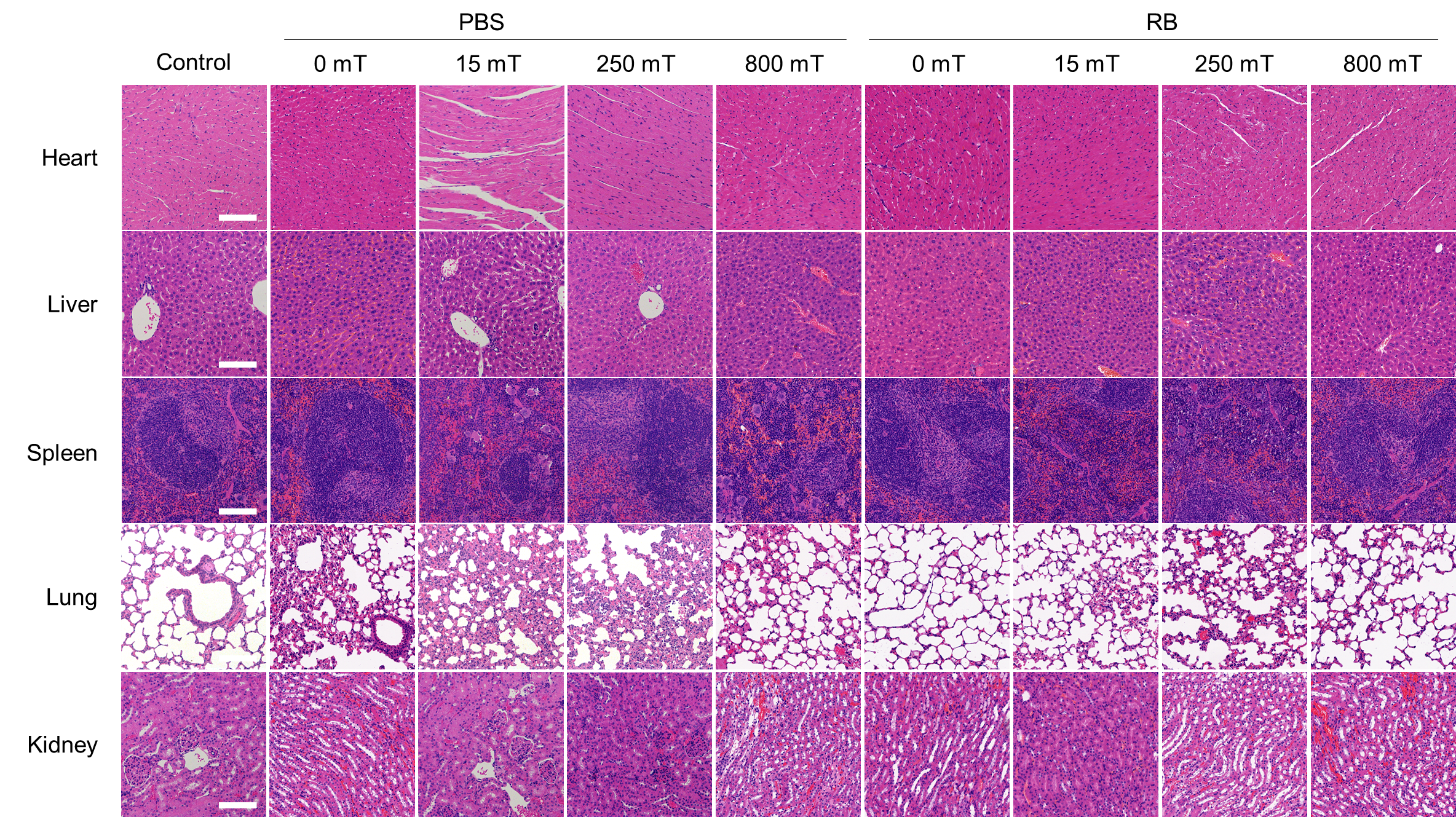


Fig. S23. H&E staining images of major organs.

Heart, liver, spleen, lung, and kidney were taken from mice in the different treatment groups. Scale bar: 100 μm.

**References**

1. Workman P, Aboagye EO, Balkwill F *et al.* Guidelines for the welfare and use of animals in cancer research. *Brit J Cancer*. 2010; **102**: 1555-77.

2. Mosinger J, Micka Z. Quantum yields of singlet oxygen of metal complexes of meso-tetrakis(sulphonatophenyl)porphine. *J Photoch Photobiol A*. 1997; **107**: 77-82.

3. Darszon A, Vandenberg CA, Schonfeld M *et al.* Reassembly of Protein-Lipid Complexes into Large Bilayer Vesicles - Perspectives for Membrane Reconstitution. *P Natl Acad Sci-Biol*. 1980; **77**: 239-43.

4. Fraga CG, Clowers BH, Moore RJ *et al.* Approach for Sample Matching of a Precursor Using Liquid Spectrometry. *Anal Chem*. 2010; **82**: 4165-73.

5. Gomolka B, Siegert E, Blossey K *et al.* Analysis of omega-3 and omega-6 fatty acid-derived lipid metabolite formation in human and mouse blood samples. *Prostaglandins Other Lipid Mediat*. 2011; **94**: 81-7.

6. Ting Hu, Cai Tie, Zhe Wang *et al.* Highly sensitive and specific derivatization strategy to profile and

quantitate eicosanoids by UPLC-MS/MS. *Anal Chim Acta*. 2017; **950**: 108-18.

7. Willenberg I, Ostermann AI, Schebb NH. Targeted metabolomics of the arachidonic acid cascade: current state and challenges of LC–MS analysis of oxylipins. *Anal Bioanal Chem*. 2015; **407**: 2675-83.

8. Hore PJ. Upper bound on the biological effects of 50/60 Hz magnetic fields mediated by radical pairs. *Elife*. 2019; **8**.
